# Molecular phylogeny of pearl oysters and their relatives (Mollusca, Bivalvia, Pterioidea)

**DOI:** 10.1186/1471-2148-10-342

**Published:** 2010-11-08

**Authors:** Ilya Tëmkin

**Affiliations:** 1 Department of Invertebrate Zoology, National Museum of Natural History, Washington, DC, USA

## Abstract

**Background:**

The superfamily Pterioidea is a morphologically and ecologically diverse lineage of epifaunal marine bivalves distributed throughout the tropical and subtropical continental shelf regions. This group includes commercially important pearl culture species and model organisms used for medical studies of biomineralization. Recent morphological treatment of selected pterioideans and molecular phylogenetic analyses of higher-level relationships in Bivalvia have challenged the traditional view that pterioidean families are monophyletic. This issue is examined here in light of molecular data sets composed of DNA sequences for nuclear and mitochondrial loci, and a published character data set of anatomical and shell morphological characters.

**Results:**

The present study is the first comprehensive species-level analysis of the Pterioidea to produce a well-resolved, robust phylogenetic hypothesis for nearly all extant taxa. The data were analyzed for potential biases due to taxon and character sampling, and idiosyncracies of different molecular evolutionary processes. The congruence and contribution of different partitions were quantified, and the sensitivity of clade stability to alignment parameters was explored.

**Conclusions:**

Four primary conclusions were reached: (1) the results strongly supported the monophyly of the Pterioidea; (2) none of the previously defined families (except for the monotypic Pulvinitidae) were monophyletic; (3) the arrangement of the genera was novel and unanticipated, however strongly supported and robust to changes in alignment parameters; and (4) optimizing key morphological characters onto topologies derived from the analysis of molecular data revealed many instances of homoplasy and uncovered synapomorphies for major nodes. Additionally, a complete species-level sampling of the genus *Pinctada *provided further insights into the on-going controversy regarding the taxonomic identity of major pearl culture species.

## Background

Since the lower Middle Ordovician some 470 million years ago, pterioidean bivalves have inhabited a remarkable diversity of marine epifaunal and semi-infaunal environments around the globe, typically confined to cryptic habitats and forming byssal attachments to various substrata. For a much shorter part of their history-from the dawn of human culture-they have become the primary source of pearls and nacre [1-3]. Pearl fishing based on natural populations of pterioidean bivalves had expanded into a growing global industry with a current value of approximately half a billion US dollars per annum [4]. In pursuit of pearls, commercial introductions and accidental transport of pterioideans beyond their native distribution ranges have greatly affected population dynamics of some species endangering local indigenous biotas (e.g., [5-8]). A recent interest in physical properties of mother-of-pearl made pterioidean bivalves a model system for elucidating molecular mechanisms of biomineralization with medical applications for bone regeneration (e.g., [9,10]). Given the long and many-sided history of pterioideans and humans, and the current economic and ecological significance of these bivalves, surprisingly little is known about the standing alpha-diversity, distribution, and evolutionary history of the group.

The present-day diversity of the superfamily Pterioidea Gray, 1847 [11] encompasses four families traditionally defined by shell shape and ligament structure: Pteriidae, Isognomonidae, Malleidae, and Pulvinitidae [12-15]. Pteriids are distinguished by an obliquely ovate shell shape, an enlarged posteriorly projecting auricle, and a deep byssal notch. Many pteriid species, particularly of genera *Pteria *and *Electroma*, evolved specialized associations with hydroid, scleractinian, and alcyonarian substrata. Most species of *Pinctada *(pearl oysters) inhabit sandy and hard bottoms, some living in association with commensal fishes and crustaceans. Several species of *Pteria *and *Pinctada *are cultured for commercial pearl production. Most species of a malleid genus *Malleus *are irregularly shaped to conform to narrow crevices in hard coral and rocky substrata, and develop elongated extensions of the hinge for stabilization in soft sediment. Species of the second malleid genus, *Vulsella*, are adapted for living within sponges. Isognomonids are characterized by the distinct morphology of the ligament that contains multiple grooves (resilifers) for ligamental attachments arranged sequentially along the hinge line (multivincular ligament). Species of the genus *Isognomon *often co-occur with species of *Malleus *in crevices and on soft muddy bottoms, but also are gregarious on mangrove roots, whereas species of *Crenatula*, the second isognomonid genus, live inside sponges (as the malleid *Vulsella*). Similarly to isognomonids, pulvinitids possess multivincular ligament, but are distinguished from isognomonids by the presence of the foramen, an opening in the right valve through which the byssus is protruded. Living pulvinitids are represented by the genus *Pulvinites*, the sole extant species of which, *Pulvinites exempla*, lives byssally attached to vertical hard substrata at 200-400 m depths.

Previous studies aimed at resolving higher-level phylogeny within Bivalvia agreed on the monophyly of the Pterioidea and its placement within the subclass Autolamellibranchiata. However, most aspects of relationships within the superfamily and the identity of its immediate sister group remain uncertain. In a pioneering study on the evolution and ontogeny of the bivalve shell, Jackson (1890) proposed the derivation of major pterioidean groups from a *Pteria*¬-like Paleozoic ancestor [16]. In his scheme, several autolamellibranchiate lineages, including common oysters of the family Ostreidae and several extinct families, evolved from *Isognomon*, the latter ultimately arising from the *Pteria *stem lineage. Jackson also provisionally recognized close relatedness of *Malleus *and *Vulsella*.

**Figure 1:**
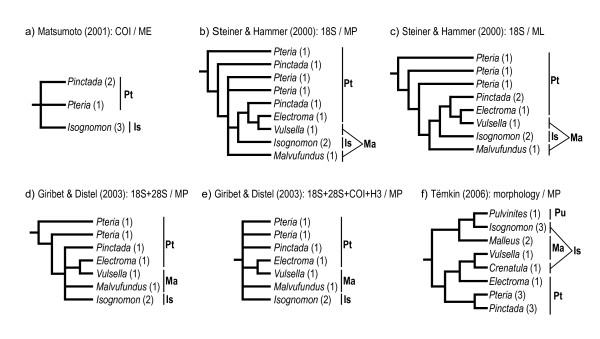
**The summary of recent hypotheses of phylogenetic relationships in the Pterioidea**. Numbers in parentheses indicate the number of terminals sampled for each taxon. Abbreviations: Is, Isognomonidae; Ma, Malleidae; ME, minimum evolution; MP, maximum parsimony; ML, maximum likelihood; Pt, Pteriidae; Pu, Pulvinitidae.

Despite the implication of Jackson's work for pterioidean systematics, the notion of the monophyly and taxonomic composition of pterioidean families persisted for over a century. Molecular studies aimed at establishing relationships within the subclass Autolamellibranchiata or the entire Bivalvia suggested that several widely accepted pterioidean taxa possibly did not constitute natural groups. The minimum evolution tree based on the analysis of the mitochondrial cytochrome oxydase I (COI) sequences did not resolve the relationships among *Pteria*, *Pinctada*, and *Isognomon *(Figure 1a) [17]. Thus, this analysis failed to provide support for the monophyly of Pteriidae but supported the monophyly of *Pinctada *and *Isognomon*. A study with more extensive sampling and based on parsimony analysis of sequence data for the nuclear small ribosomal subunit (18S rDNA) likewise failed to recover the monophyly of Pteriidae, but also that of Malleidae (Figure 1b) [18]. In that study, *Isognomon *was monophyletic, *Pteria *was paraphyletic, and *Pinctada *was polyphyletic. The pteriid *Electroma *was a sister taxon to the malleid *Vulsella*. The maximum likelihood analysis of the same data produced a similar, but more resolved, topology recovering monophyletic *Pinctada *(Figure 1c) [18]. In a more extensive in character-but not taxonomic-sampling analysis of combined 18S and the large ribosomal subunit sequence (28S rDNA) data, the families Pteriidae and Malleidae, and the genus *Pteria *were non-monophyletic (Figure 1d) [19]. When these data were combined with the nuclear histone H3 gene and the mitochondrial COI gene sequences, the analysis produced a polytomy for all representatives of the Pterioidea except for the *Isognomon *and *Electroma*/*Vulsella *clades (Figure 1e) [19].

In summary, molecular studies, varying greatly in sampling and analytical techniques, produced inconsistent hypotheses of pterioidean relationships with low resolution and support. These findings, however, agreed on the monophyly of the superfamily and suggested that the families Pteriidae and Malleidae were not monophyletic. The results were conflicting with regard to the monophyly of the genera *Pteria *and *Pinctada*, whereas *Isognomon *was invariably monophyletic. The monophyly of the family Isognomonidae and the genera *Electroma *and *Malleus *were not tested, and representatives of the family Pulvinitidae and the genus *Crenatula *were not included in the analyses. The only study that focused exclusively on the Pterioidea was based on morphological characters and included representatives of all four families and nine genera (Figure 1f) [15]. It supported the monophyly of the Pterioidea and non-monophyly of three families (Pulvinitidae remains untested being represented by a single extant species). All the genera that were represented by multiple species (*Pteria*, *Pinctada*, *Isognomon*, and *Malleus*) were recovered monophyletic. Species-level sampling in that study was insufficient to test the assumption of monophyly of the remaining genera and did not include type species for most nominal supraspecific taxa. Despite these recent challenges on both molecular and morphological grounds, no consensus on pterioidean relationships was reached and no revision of the existing classification was proposed.

The identity of the pterioidean sister group remains uncertain, favoring the oysters (Ostreoidea), pen shells (Pinnoidea), or a clade composed of these two groups, currently classified together with the Pterioidea in the order Pterioida. Most analyses based on morphological data for extinct, extant, or both kinds of taxa, have suggested a sister relationship of the Pterioidea and Pinnoidea [20-24], except for one, which instead suggested a closer relationship of the Pterioidea and Ostreoidea [15]. Likewise, molecular analyses were divided in favoring either Pinnoidea [25] or Ostreoidea [18,19,26] as a sister group of the Pterioidea. In other studies, either Pinnoidea and Ostreoidea formed a clade sister to the Pterioidea [17,19], or the relationships among the three superfamilies were unresolved [25,27]. The only explicitly combined analysis of morphological and molecular data resolved the Pinnoidea basal to the Pterioidea/Ostreoidea clade [24]. The unsettled question of the pterioidean sister group is pertinent to the recent debates on the origin of oysters in paleontological literature [28-30].

The present analysis is the first study to produce a nearly comprehensive species-level phylogeny for the Pterioidea based on DNA sequence data. The principal specific goals of this study include (1) testing the monophyly of the Pterioidea, and all its families and genera; (2) reviewing the consequences of these results for the homology of important morphological characters; and (3) resolving species-level phylogenetic relationships within the commercially important pearl oyster genus *Pinctada*.

## Methods

### Taxonomic Sampling

#### Sources of specimens

Most specimens were obtained from museum collections worldwide, with additional material gathered during fieldwork in the western Atlantic (Florida Keys, U.S.A., June 2003), the eastern Pacific (Golfo de California, Mexico, August 2003), the Mediterranean (Tyrrhenian Sea, Italy, May 2005), and the Indo-Pacific (Gulf of Thailand, Thailand, August-September 2005) regions. Living animals were collected in mangroves and shallow intertidal waters, fixed in 85-95% ethanol, and subsequently transferred to 75-95% ethanol. Collected specimens were deposited at the American Museum of Natural History (New York, U.S.A.) and the Field Museum of Natural History (Chicago, U.S.A.). DNA sequence data were obtained for 77 of the 100 terminal taxa. The remaining sequences were acquired from GenBank http://www.ncbi.nlm.nih.gov/Genbank/index.html. Collection localities and museum catalog numbers for the specimens used in this study are listed in Table 1.

**Table 1:** Specimen and DNA sequence sources.

Taxon	Voucher ID	GenBank Acc. No. (18S/28S/16S/H3)	Locality data
Mytiloidea			
*Mytilus edulis*	AMNH 107223	L33448/AF120587/AY484747/AY070150	NA

Pectinoidea			
*Argopecten irradians*	AMNH 107223	L11265/AY145391/HQ329396/HQ329247	NA
*Pecten jacobaeus*		AY070112/AY070125/AJ245394/AY070153	NA

Pinnoidea			
*Atrina rigida*	AMNH 305138	HQ329323/HQ329438/HQ329397/HQ329248	Florida Keys, USA
*Atrina seminuda*	AMNH 305162	HQ329324/HQ329439/HQ329398/HQ329249	Florida Keys, USA
*Pinna carnea*	AMNH 305177	HQ329375/HQ329489/HQ329431/HQ329302	Florida Keys, USA
*Pinna muricata*		AJ389636/AB102754/NA/NA	NA

Ostreoidea			
*Ostrea edulis*		L49052/Z29551/AF540597/AY070151	NA
*Crassostrea virginica*	AMNH 319315	X60315/AF137050/AY905542/HQ329250	NA
*Crassostrea gigas*		AB064942/Z29546/AF280611/NA	NA
*Hyotissa hyotis*	FMNH 302010	AJ389632/AF137036/AY548883/HQ329258	NA
*Hyotissa numisma*		AJ389633/AF137035/AY376598/NA	NA

**Pterioidea**			
Pteriidae			
*Pinctada albina *1	AMNH 319311	HQ329358/HQ329472/HQ329415/HQ329286	Western Australia
*Pinctada albina *2		AB214453/AB214468/AB214438/NA	The Amami Islands, Japan
*Pinctada albina *3	AMNH 319342	HQ329370/HQ329484/HQ329426/HQ329297	Northern Territory, Australia
*Pinctada capensis *1	NMP W3373a	HQ329359/HQ329473/HQ329416/HQ329287	South Africa
*Pinctada capensis *2	NMP W3373b	HQ329360/HQ329474/HQ329417/HQ329288	South Africa
*Pinctada capensis *3	NMP W3374	HQ329361/HQ329475/HQ329418/HQ329289	South Africa
*Pinctada chemnitzii*		AB214452/AB214467/AB214437/NA	Myanmar
*Pinctada fucata *1		AB214463/AB214478/AB214448/NA	The Amami Islands, Japan
*Pinctada fucata *2		AB214462/AB214477/AB214447/NA	Kamikoshiki island, Japan
*Pinctada fucata *3		AB214461/AB214476/AB214446/NA	Hainan, China
*Pinctada fucata *4		AB214460/AB214475/AB214445/NA	Cambodia
*Pinctada fucata *5		AB214459/AB214474/AB214444/NA	Western Australia
*Pinctada fucata *6		AB214458/AB214473/AB214443/NA	Myanmar
*Pinctada fucata *7	AMNH 319310	HQ329362/HQ329476/HQ329419/NA	Japan [pearl farm]
*Pinctada fucata *8	AMNH 319278	HQ329363/HQ329477/HQ329420/HQ329290	Chantaburi, Thailand
*Pinctada fucata *9	AMNH 319222	HQ329373/HQ329487/HQ329429/HQ329300	Western Australia
*Pinctada fucata *10		AB214464/AB102763/AB214449/NA	Anamizu, Japan
*Pinctada imbricata *1	AMNH 319321	HQ329364/HQ329478/HQ329421/HQ329291	Guayacan, Venezuela
*Pinctada imbricata *2	AMNH 305121	HQ329365/HQ329479/HQ329422/HQ329292	Florida Keys, USA
*Pinctada imbricata *3		AB214456/AB214471/AB214441/NA	Florida Keys, USA
*Pinctada imbricata *4	AMNH 308116	HQ329366/HQ329480/HQ329423/HQ329293	Florida Keys, USA
*Pinctada longisquamosa *1	AMNH 298905	HQ329367/HQ329481/NA/HQ329294	Florida Keys, USA
*Pinctada longisquamosa *2	AMNH 308234	HQ329368/HQ329482/HQ329424/HQ329295	Florida Keys, USA
*Pinctada maculata*		AB214455/AB214470/AB214440/NA	The Amami Islands, Japan
*Pinctada margaritifera *1	AMNH 319369	HQ329369/HQ329483/HQ329425/HQ329296	Florida, USA
*Pinctada margaritifera *2		AB214451/AB214466/AB214436/NA	Okinawa, Japan
*Pinctada maxima*		AB214450/AB214465/AB214435/NA	Philippines
*Pinctada mazatlanica*	AMNH 311790	HQ329371/HQ329485/HQ329427/HQ329298	Sonora, Mexico
*Pinctada nigra *1	AMNH 319354	HQ329372/HQ329486/HQ329428/HQ329299	South Africa
*Pinctada nigra *2		AB214454/AB214469/AB214439/NA	The Amami Islands, Japan
*Pinctada nigra *3	AMNH 319334	HQ329374/HQ329488/HQ329430/HQ329301	Northern Territory, Australia
*Pinctada radiata*		AB214457/AB214472/AB214442/NA	NA
*Pteria avicular *1	MNHN 42746	HQ329382/HQ329493/NA/HQ329306	Bohol Island, Philippines
*Pteria avicular *2	AMNH 319350	HQ329388/HQ329498/HQ329434/HQ329311	Bay of Bengal, India
*Pteria avicular *3	MNHN 42752	HQ329392/HQ329506/NA/HQ329319	Solomon Islands
*Pteria avicular *4	MNHN 42749	HQ329389/HQ329503/NA/HQ329316	Bohol Island, Philippines
*Pteria avicular *5	AMNH 319286a	HQ329376/HQ329490/NA/HQ329303	Chantaburi, Thailand
*Pteria avicular *6	AMNH 319286b	HQ329377/HQ329491/NA/HQ329304	Chantaburi, Thailand
*Pteria colymbus *1	AMNH 319352	HQ329384/HQ329494/HQ329432/HQ329307	Bahamas
*Pteria colymbus *2	UMML 30.11787	HQ329385/HQ329495/NA/HQ329308	Caribbean Sea
*Pteria hirundo*	AMNH 319357	HQ329383/HQ329497/HQ329433/HQ329310	Mediterranean Sea, Italy
*Pteria howensis *1	MNHN 42750	HQ329390/HQ329504/NA/HQ329317	Bohol Island, Philippines
*Pteria howensis *2	MNHN 42751	HQ329391/HQ329505/NA/HQ329318	Bohol Island, Philippines
*Pteria howensis *3	NMNZ M160878	HQ329393/HQ329507/NA/HQ329320	Norfolk Island, Australia
*Pteria lata *1	AMNH 319337	HQ329379/HQ329499/HQ329435/HQ329312	Darwin Harbour, Australia
*Pteria lata *2	AMNH 319286c	HQ329378/HQ329492/NA/HQ329305	Chantaburi, Thailand
*Pteria loveni*	MNHN 42747	HQ329380/HQ329500/HQ329436/HQ329313	Solomon Islands
*Pteria penguin *1	MNHN 42748	HQ329381/HQ329501/NA/HQ329314	Panglao Island, Philippines
*Pteria penguin *2	AMNH 319290	HQ329386/HQ329496/NA/HQ329309	Chantaburi, Thailand
*Pteria sterna*	AMNH 311731	HQ329394/HQ329508/NA/HQ329321	Guaymas, Mexico
*Pterelectroma physoides*	AMNH 319319	HQ329387/HQ329502/HQ329437/HQ329315	Raffles Bay, Australia
*Electroma alacorvi *1	AMNH 292446	HQ329326/NA/HQ329400/HQ329252	Cocos-Keeling
*Electroma alacorvi *2	MNHN 42753	HQ329328/HQ329442/HQ329401/HQ329254	Balicasag Island, Philippines
*Electroma *cf. *alacorvi *1	AMNH 319220	NA/HQ329443/HQ329402/HQ329255	Houtman Abrolhos, Australia
*Electroma *cf. *alacorvi *2	AMNH 319219	HQ329329/HQ329444/HQ329403/HQ329256	Houtman Abrolhos, Australia
*Electroma *cf. *alacorvi *3	AMNH 319221	HQ329330/HQ329445/HQ329404/HQ329257	Houtman Abrolhos, Australia
*Electroma papilionacea*	AMNH 319340	HQ329327/HQ329441/NA/HQ329253	Raffles Bay, Australia
Isognomonidae			
*Isognomon alatus*	AMNH 305129	HQ329331/HQ329446/HQ329405/HQ329259	Florida Keys, USA
*Isognomon bicolor*	AMNH 307896	HQ329332/HQ329447/HQ329406/HQ329260	Florida Keys, USA
*Isognomon californicum*	CASIZ 112485	HQ329333/HQ329448/NA/HQ329261	Oahu, Hawaii
*Isognomon ephippium*	AMNH 319336	HQ329334/HQ329449/NA/HQ329262	Darwin Harbor, Australia
*Isognomon *cf. *ephippium *A1	AMNH 319294b	HQ329341/HQ329456/NA/HQ329269	Koh Nam Sao Island, Thailand
*Isognomon *cf. *ephippium *A2	AMNH 319292b	HQ329343/HQ329458/NA/HQ329271	Koh Nam Sao Island, Thailand
*Isognomon *cf. *ephippium *B	AMNH 319253	HQ329335/HQ329450/HQ329407/HQ329263	Kungkrabaen Bay, Thailand
*Isognomon isognomum *1	AMNH 319283	HQ329336/HQ329451/NA/HQ329264	Chong Saba Island, Thailand
*Isognomon isognomum *2	LACM 85-2	HQ329347/HQ329462/NA/HQ329275	Phuket, Thailand
*Isognomon isognomum *3	MNHN 42754	HQ329339/HQ329454/NA/HQ329267	Panglao Island, Philippines
*Isognomon isognomum *4	AMNH 319294a	HQ329340/HQ329455/NA/HQ329268	Koh Nam Sao Island, Thailand
*Isognomon isognomum *5	AMNH 319292a	HQ329342/HQ329457/NA/HQ329270	Koh Nam Sao Island, Thailand
*Isognomon isognomum *6	FMNH 311977	HQ329344/HQ329459/NA/HQ329272	Kungkrabaen Bay, Thailand
*Isognomon isognomum *7	AMNH 319260	HQ329337/HQ329452/NA/HQ329265	Kungkrabaen Bay, Thailand
*Isognomon radiatus*	AMNH 305142	HQ329338/HQ329453/HQ329408/HQ329266	Florida Keys, USA
*Isognomon spathulata*	AMNH 319257	HQ329348/HQ329463/NA/HQ329276	Kungkrabaen Bay, Thailand
*Isognomon *sp. A1	AMNH 319230	HQ329345/HQ329460/NA/HQ329273	Houtman Abrolhos, Australia
*Isognomon *sp. A2	CASIZ 104281	HQ329346/HQ329461/HQ329409/HQ329274	Clipperton Island
*Crenatula avicularis*	USNM 795306	HQ329325/HQ329440/HQ329399/HQ329251	Noumea, New Caledonia
Malleidae			
*Malleus albus*	AMNH 319271	HQ329350/HQ329464/NA/HQ329278	Kungkrabaen Bay, Thailand
*Malleus *cf. *albus *1	AMNH 319298	HQ329349/NA/HQ329410/HQ329277	Rottnest Island, Australia
*Malleus *cf. *albus *2	AMNH 319225	HQ329356/HQ329470/NA/HQ329284	Houtman Abrolhos, Australia
*Malleus *cf. *albus *3	AMNH 319226	HQ329357/HQ329471/NA/HQ329285	Houtman Abrolhos, Australia
*Malleus candeanus*	AMNH FK-685	HQ329351/HQ329465/HQ329411/HQ329279	Florida Keys, USA
*Malleus malleus*	MNHN 42755	HQ329352/HQ329466/HQ329412/HQ329280	Bohol Island, Philippines
*Malleus regula *1	AMNH 319335	HQ329353/HQ329467/HQ329413/HQ329281	Darwin Harbor, Australia
*Malleus regula *2	AMNH 319339	HQ329354/HQ329468/HQ329414/HQ329282	Darwin Harbor, Australia
*Malleus regula *3	MNHN 42756	HQ329355/HQ329469/NA/HQ329283	Panglao Island, Philippines
*Vulsella vulsella*	AMNH 319281	HQ329395/HQ329509/NA/HQ329322	Kungkrabaen Bay, Thailand
*Vulsella *cf. *vulsella*		AJ389642/AB102765/NA/NA	NA
Pulvinitidae			
*Pulvinites exempla*	NMNZ M150090	AJ414640/NA/NA/NA	East Cape, New Zealand

Museum collection acronyms: AMNH, American Museum of Natural History, New York; CAS, California Academy of Sciences, San Francisco; FMNH, Field Museum of Natural History, Chicago; LACM, Los Angeles County Museum; MNHN, Muséum National d'Histoire Naturelle, Paris; NMP, Natal Museum, Pietermaritzburg; USNM, National Museum of Natural History, Smithsonian Institution, Washington, D. C. [= United States National Museum]; UMML, Rosenstiel School of Marine and Atmospheric Science, University of Miami, Florida [=University of Miami Marine Laboratory]. NA, not available.

#### Outgroup taxa

Given that the Pterioidea is currently accepted as monophyletic and the Pinnoidea or Ostreoidea were established as the taxa most closely related to the Pterioidea on morphological and molecular grounds, representatives of these superfamilies were chosen for the outgroup. To ensure maximum diversity of the ostreoidean taxa, the selection of the gryphaeid *Hyotissa*, and the ostreid *Ostrea *and *Crassostrea *exemplars were guided by their placement in a recently proposed phylogenetic hypothesis [31]. To polarize the immediate outgroups, representatives of two other autolamellibranchiate orders, Mytiloida and Pectinoida were selected based upon the availability of sequence data. Mytilioda, represented by the well-known common mussel species, *Mytilus edulis*, had been resolved as the most basal order of the subclass in comprehensive studies based on morphological [22], molecular [18,24], and combined [24] data and, accordingly, was designated for cladogram rooting.

#### Ingroup taxa

Inasmuch as the Pterioidea is the primary focus of this study, the taxonomic sampling of this taxon was the most dense. All nominal families and genera of the Pterioidea were included in the analyses. The sample contained representatives of type species of all pterioidean genera except for *Pulvinites*, which was described based on an extinct species, *P. adansonii*, from the Late Cretaceous. Success in securing multiple representatives for each genus varied because the material could not be obtained for few taxa characterized by cryptic life habit and presumed low species diversity. Initially, specimens were sorted to morphospecies using anatomical and shell morhological features. Species-level identifications were verified with reference to the extant type material and assigned to most specimens. Where possible, to avoid assumptions of the extent of intraspecific phenotypic variation, multiple potentially conspecific individuals were included in the analysis. Given the lack of recent systematic revisions for the majority of pterioidean taxa, species-level identifications were tentatively chosen with correspondence to their prevailing use in current literature.

The sample of the genus *Pinctada *was complete, with 32 exemplars representing 10 nominal species, including the type species *Pinctada margaritifera*. The overrepresentation of *Pinctada *species was necessitated by an enduring controversy regarding the taxonomic status of the *Pinctada imbricata*/*fucata*/*radiata *species complex [32] that includes important perliculture species. Sampling of *Pteria *was nearly comprehensive; it included 18 exemplars of 10 nominal species (including the type species *Pteria hirundo*) of estimated total of 12 species. The genus *Isognomon *was represented by 18 exemplars corresponding to potentially 10 of approximately 13 species total, and includes the type species, *I. isognomum*. The genus *Malleus *was represented by 9 exemplars, corresponding to 5 (of total approximately 8) species, including *Malleus malleus*, the type species of the genus, and *Malleus regula*, a type species of the subgenus *Malvufundus*. It must be pointed out that the true global species diversity of *Isognomon *and *Malleus *is not known due to potential high endemism and cryptic diversity associated with adaptations to hidden habitats, such as interstitial crevices and submarine caves. The extent of species coverage for the relatively rare and inadequately studied genera *Vulsella, Crenatula*, and *Electroma *is uncertain due to cryptic lifestyle in coral crevices and inside sponges. A broad survey based on dry shells from over twenty major museum collections worldwide suggested that these genera contain very few species and that *Crenatula *is likely to be monotypic. The latter was represented in the analysis by a single specimen corresponding to the type species, *C. avicularis *(previously used in the morphology-only phylogenetic analysis under the synonym *C. modiolaris *[15]). The genus *Electroma *was represented by seven exemplars corresponding to potentially four species, including *E. alacorvi *and *Electroma *(*Pterelectroma*) *physoides *[commonly referred to by its junior synonym, *E. (P.) zebra*], the sole living member of the subgenus. Two exemplars of *Vulsella*, corresponding to the type species *V. vulsella*, one of possibly only two living species, were included in the analysis. The genus *Pulvinites *was represented by one exemplar of the only extant pulvinitid species, *P. exempla*.

### Molecular Data

#### Locus selection

Genome-wide sampling of multiple independently evolving genes is instrumental in overcoming the incongruence among trees derived from individual gene analyses, and producing a resolved and strongly supported phylogeny. Because this study involves the analysis of taxa of disparate levels of divergence, a data set was assembled that consisted of nuclear loci (two ribosomal RNA genes and the histone H3) and a mitochondrial ribosomal RNA locus, that in combination span a broad spectrum of variation. The ribosomal DNA sequences display a substantial difference in evolutionary rates among and within the genes (from fast-evolving mitochondrial to the much more conserved nuclear rDNA), and are easily amplifiable due to the presence of multiple copies per genome and the pattern of concerted evolution [33]. The following DNA regions were used: (1) the complete sequence of the small nuclear ribosomal subunit (SSU or 18S rDNA; range 1237-1778 bp; total aligned length 1882 bp); (2) the D1-D3 fragments of the large nuclear ribosomal subunit (LSU or 28S rDNA; range 308-1131 bp; total aligned length 1308 bp); (3) a fragment of the large mitochondrial ribosomal subunit (mtLSU or 16S rDNA; range 466-580 bp; total aligned length 934 bp), and (4) the single-copy nuclear histone gene H3 (the total length 310 bp), which is intron-free and highly conserved at the level of amino acids. The total length of the alignment of the complete data set was 4434 bp.

#### DNA isolation and amplification

Original DNA sequence data have been obtained for most sampled terminals; remaining sequences were obtained from the GenBank database (Table 1). DNA was extracted from fresh, frozen, dried, ethanol- (70-100%) or RNAlater^®^(Qiagen)-preserved tissues. Total genomic DNA was isolated from adductor muscle, foot, or the entire body using DNeasy^® ^Tissue Kit (Qiagen). DNA amplifications were carried out using GeneAmp^® ^PCR System 9700 (Applied Biosystems) using standard procedures. The PCR primers are listed in Table 2. Amplifications were carried out in 35-40 cycles using the following general temperature profile: initial denaturation for 5 min at 94°C, denaturation for 30-45 s at 94°C, annealing for 15-60 s at 40-65°C, and extension for 30-120 s at 72°C, with a final extension at 72°C for 5 min. Specific conditions varied across loci and taxa. The PCR products were subsequently desalted and concentrated using an ArrayIt^® ^PCR Product Purification Kit (TeleChem International) on a Biomek^® ^2000 Laboratory Automation Workstation (Beckman Coulter), and sequenced in both directions using the ABI Prism^® ^BigDye^™ ^Terminator Cycle Sequencing Reaction Kit (Applied Biosystems) on ABI Prism^® ^3700/3730xl DNA Analyzers (Applied Biosystems) at AMNH.

**Table 2:** Sources and sequences of forward (F) and reverse (R) primers.

Locus	Primer	Sequence, 5' - 3'	Source
18S	1F (F)	TACCTGGTTGATCCTGCCAGTAG	[162]
	3F (F)	GTTCGATTCCGGAGAGGGA	[162]
	5R (R)	CTTGGCAAATGCTTTCGC	[162]
	9R (R)	GATCCTTCCGCAGGTTCACCTAC	[162]
	A20 (F)	ATGGTTGCAAAGCTGAAAC	[162]
	S2 (F)	GAGTAAATTAGAGTGTTCAAAGCA	[162]
	S3 (R)	CGGAATTAACCAGACAAATC	[162]
28S	D1F (F)	GGGACTACCCCCTGAATTTAAGCA	[163]
	D6R (R)	CCAGCTATCCTGAGGGAAACTTCG	[163]
16S	16SaR (F)	CGCCTGTTTATCAAAAACAT	[164]
	16SbR (R)	CCGGTCTGAACTCAGATCACGT	[164]
	16SmasF (F)	CGCCTGGTTGATTAAAAACATTGCTGC	[161]
	16SmasR (R)	CCGGTTTGAACTCAGATCACGTA	[161]
H3	H3aF (F)	ATGGCTCGTACCAAGCAGACVGC	[165]
	H3aR (F)	ATATCCTTRGGCATRATRGTGAC	[165]

Sequence fragments were assembled into contigs, and checked for errors and ambiguities against chromatograms using Sequencher™ 4.6 (Gene Codes). The sequence coverage varied from 2× to 6× depending on the quality of the material. The resultant sequences were checked for potential contamination by BLAST searches [34,35] as implemented by the NCBI website http://www.ncbi.nlm.nih.gov. The new sequences have been deposited in GenBank under accession numbers HQ329247-HQ329509 (Table 1).

#### DNA sequence homology

The ribosomal DNA sequences, the principal source of character data in the present study, vary considerably in length, creating uncertainty in the inference of nucleotide-level homology. To circumvent alignment ambiguity, all potential nucleotide homologies were evaluated in the framework of dynamic homology [36,37], as implemented in POY 4.1 [38]. In this approach nucleotide correspondences (putative positional homologies) and their transformations (substitutions and insertion-deletion events) are simultaneously optimized for each tree topology provided an optimality criterion and a cost function (transformation step matrix). The integration of alignment and tree searching into a single procedure to produce globally optimal trees allows for evaluating multiple unique homology schemes on a consistent theoretical basis, as both are evaluated using the same criteria [39,40]. In simultaneous analysis using direct optimization, morphological and molecular characters are co-optimized at the level of sequence alignment in search of a globally optimal solution. The homology scheme corresponding to the topology of the optimal cladogram was used to produce an implied alignment [41,42]. The implied alignment was used for statistical tests of sequence variation and other analyses requiring fixed character matrices with equal length character strings (*i.e*. multiple alignments).

DNA sequences were partitioned into fragments to reduce computation time and to constrain the assignment of insertions and deletions (indels) to unambiguously homologous regions. None of the regions were excluded from the analyses except for the flanking primer sequences. Initially, multiple alignments were constructed using ClustalX 2.0.5 [43] under default parameter settings. For rDNA sequences, the alignments were subsequently partitioned into fragments flanked by highly conservative (lacking indels) regions corresponding to elements of secondary structure. The secondary structure features were identified with reference to those inferred for molluscan [44,45] and other metazoan (e.g., [46-50]) taxa. Lastly, the gaps were removed resulting in a total of 26 matrices of unaligned fragments. All histone H3 sequences had the same length. A comparison of the translated H3 open reading frame with the published database of histone sequences [51] confirmed high conservation at the level of amino acids. In this case, the nucleotide homology was unambiguous and the H3 data set was treated as a single aligned fragment.

#### Patterns of nucleotide substitutions

Individual data partitions were tested for substitution saturation using a non-parametric statistical test based on an information entropy index [52] implemented in DAMBE 5.0.23 [53]. The actual number of transitions and transversions, and their ratio were obtained by optimizing substitutions on the optimal topology using MacClade 4.07 [54]. Departure from homogeneity of base composition across all taxa was assessed using the χ^2 ^test as implemented in PAUP* 4.0d105 [55]. The extent of rate variation across sites for individual data partitions and for the entire data set was estimated by the shape parameter α of the gamma distribution. The values of α were estimated by the maximum likelihood method assuming the discrete gamma model [56,57] with four rate categories as implemented in PAUP* 4.0d105 [55].

For close inspection of species-level divergence of the problematic *Pinctada imbricata*/*fucata*/*radiata *species complex, the genetic distances were obtained for within and among presumed species, and then compared to the inter- and intraspecific levels of sequence divergence inferred for their congeners. In this analysis, the distance matrices for rDNA data sets were generated using the minimum evolution objective function (ME; [58,59]) under the general time-reversible model of nucleotide substitution (GTR; [60-62]) using PAUP* 4.0d105 [55]. The choice of the substitution model was guided by the best-fit model preferred by the Akaike Information Criterion (AIC; [63]) for the combined molecular data set under maximum likelihood (ML) optimality criterion (see below). The H3 data set was not used in this analysis due to missing data for several critical taxa in question.

### Morphological Data

A previously published data set based on morphological data [15] was used for simultaneous analysis of morphological and molecular data. The morphological characters and methods have been described and illustrated in detail in [15]. The data set included 134 morphological characters scored for 19 exemplar species, representing all valid pterioidean genera. As in the original study, all characters were unweighted and one multistate character (the differentiation of gill filaments) was treated as additive based on unequivocal ontogenetic evidence. Molecular data were available for all the taxa for which morphological characters were sampled with the exception of *Malleus anatinus*. Therefore, for the simultaneous analysis of morphological and molecular data sets, the sequences of a closely related species, *M. albus*, were used as a surrogate for the missing molecular data for *M. anatinus*. Given the small number of taxa for which morphological data was available, the benefits of minimizing the amount of missing data at the expense of using a composite taxon was justified in the context of the present analysis aimed primarily at resolving supraspecific relationships. For the morphological character matrix, see [15].

### Character Matrices and Incongruence-Length Difference Tests

Several different molecular data sets varying in taxon and character sampling were examined in the present study. The most comprehensive character matrix (hereafter referred to as the "complete data set") included sequences for four loci scored for 100 exemplars (12 outgroup and 88 ingroup terminal taxa). The complete data set contained representatives of all supraspecific taxa and approximately 92% of all extant species of the Pterioidea. In this data set 43% terminals were represented by sequences for all four loci, 54%-by three loci, 2%-by two loci, and only 1%-by a short fragment of a single locus. Thus, character sampling was not satisfactory for 3% of terminal taxa (the ingroup taxa *Pulvinites exempla *and *Vulsella vulsella*, and the outgroup taxon *Pinna muricata*), and their placement must therefore be treated with caution. The second character matrix (hereafter referred to as the "reduced data set") was restricted to the subset of those taxa for which morphological data was available. The reduced data set was tailored for a combined analysis to have a maximum taxonomic overlap of morphological and molecular character matrices with minimum missing data. This data set contained 19 exemplars (3 outgroup and 16 ingroup terminal taxa), representing all pterioidean genera and families. Morphological data was scored for all 19 terminals; 14 of these terminals had a complete complement of molecular data (4 loci), 4 terminals had sequence data for three loci, and one terminal (*Pulvinites exempla*) was represented by a single fragment of the 18S locus. The simultaneous analyses of molecular and morphological data were performed under maximum parsimony optimality criterion with the same cost for transformations of morphological characters as for nucleotide substitutions. In addition to the complete and reduced matrices of combined molecular data, five data sets corresponding to the combined nuclear data (18S, 28S, and H3) and the four individual loci (18S, 28S, 16S rDNA, and histone H3) were analyzed in the parsimony framework to explore topological differences, and the extent of resolution and support among the partitions.

To evaluate the degree of incongruence between the data sets quantitatively, the incongruence-length difference tests (ILD; [64-66]) were performed in PAUP* 4.0d105 [55] [but see, e.g., [67,68] for criticisms of the ILD test]. The ILD tests were done simultaneously on the four individual locus matrices as well as pairwise on the complete data set. The tests were performed under parsimony using 100 replicates, where each replicate consisted of 10 random-addition sequences (RAS), with one tree retained per iteration, followed by tree bisection and reconnection (TBR; [69]) branch swapping to completion.

### Phylogenetic Analyses

#### Tree searching and optimization

Phylogenetic analyses of DNA sequence data were performed using direct optimization [70], a heuristic approximation to the optimal tree alignment method [71-75], under parsimony optimality criterion. A heuristic extension of generalized optimization [76,77], direct optimization includes insertion and deletion events in addition to substitutions as character transformations, allowing for the analysis of sequences of different lengths. Additional analyses based on a static alignment and ML optimality criterion were performed to characterize the patterns of nucleotide substitutions and to explore potential sources of systematic error for inferences based on the assumptions of parsimony. These analyses were based on the implied alignment corresponding to the single optimal cladogram obtained from the parsimony analysis of the combined data performed by direct optimization under the equal-cost regime for the combined molecular character data set.

Parsimony analyses under direct optimization, as implemented in POY 4.1 [38], were carried out in parallel on the American Museum of Natural History Parallel Computing Cluster and a dual-processor personal computer. A stepwise tree searching strategy that combines independent multiple starting points and efficient algorithms designed for escaping local optima was used to insure sufficient sampling of tree space. Initially, 250-300 Wagner trees [78,79] were generated by RAS and submitted to branch swapping to completion by alternating rounds of subtree pruning and regrafting (SPR; [69]) and TBR. The resultant optimal cladograms were subjected to 50 iterations of TBR parsimony ratchet [80], upweighting 20% of nucleotide characters by a factor of 5 and retaining one optimal cladogram per iteration. In the next step, the optimal cladograms were submitted to tree fusing [81], allowing for up to 200 pairwise clade exchanges, which was followed by another round of TBR. Finally, the resultant optimal cladograms were submitted to a final round of TBR under exact three-dimensional direct optimization (iterative pass optimization; [82]), a more efficient, but substantially more computationally intensive method. The final equally most-parsimonious trees (MPTs) were rediagnosed to ensure the correctness of tree length calculation and were used to infer implied alignment. All tree searches were performed with unconstrained ingroup and outgroup designation [83].

The ML analyses were performed using RAxML 7.0.4 [84] for 250 independent replicates. The results were evaluated for topological congruence and convergence on the optimal likelihood value to ensure that the heuristic searches of tree space were sufficiently thorough. There appears to be no starting-point dependence to successive approximation that could potentially compromise the accuracy of approximation compared to full-optimization of ML searches [85]. The best-fit model of substitution, given the optimal cladogram and the corresponding implied alignment, was selected by Modeltest 3.7 [86] for the combined molecular data set using the Akaike Information Criterion (AIC; [63,87]). The model favored by the Akaike Information Criterion (AIC = 60431.5938) corresponded to the general time-reversible model that included estimation of the proportion of invariant sites (P-Invar) and assumed a gamma distributed rate variation among sites (GTR + I + Γ). However, due to non-independence of P-Invar and the shape parameter α of the gamma distribution [88,89], the ML analyses were performed under the GTR + Γ model. Topological differences between the trees based on the partitioned and combined data do not present a problem for choosing among alternative models of sequence evolution, because tree topology does not strongly affect model estimation unless long internal branches are involved [90].

The ensemble consistency index (CI; [91]) and the ensemble retention index (RI; [78,91]) for the optimal tree(s) were calculated in POY 4.1 [38] with the dynamic-homology characters transformed into static nucleotide-level characters by static approximation [41]. The robustness of phylogenetic relationships was evaluated using the Bremer (BrS; [92]) and jackknife (JK; [93]) support methods at nucleotide level. Relative contribution of individual genes was assessed by partitioned Bremer support (PBrS; [94]). The batch files for constrained searches were generated by TreeRot 3 [95]. The constrained searches (100 replicates per search) were performed by PAUP* 4.0d105 [55]. For calculating jackknife support values, 2000 resampling iterations with 36% of sites removed during each pseudoreplicate were performed using POY 4.1 [38]. For the partitioned analyses, only jackknife support values were estimated.

The resulting trees were visualized using POY 4.1 [38] and FigTree 1.2.2 [96]. The optimization and ancestral state reconstruction of morphological characters were evaluated using MacClade 4.07 [54].

The significance of the length difference between alternative topologies was tested by the Kishino-Hasegawa test using resampling of estimated log-likelihoods (RELL) bootstrap (the KH test; [97,98]) with 1000 replicates under ML criterion, and the Wilcoxon signed-ranks test (the Templeton test; [99,100]) and the KH test under parsimony criterion as implemented in PAUP* 4.0d105 [55]. The tests conducted under ML were performed using the GTR + Γ, the same model used for the ML tree searches. The likelihood-based tests are generally considered inapplicable for post-hoc tests of the optimal versus an alternative topology [101,102]. In the present study, these paired-sites tests were used to assess the significance of alternative outgroup taxon configurations using constrained topologies defined *a priori *(none of which corresponding to the actual optimal tree) for the reduced molecular data set.

#### Indel treatment and sensitivity analysis

Because direct optimization integrates the implied alignment and the explicit topology, the choice of analytical parameters (character state transformation weights, or costs, for nucleotide substitutions and indels) is critical for phylogenetic inference, as analyses performed under alternative cost matrices can produce widely different hypotheses of relationships. To evaluate the effect of varying alignment parameter values on cladogram topology, independent analyses of the complete data set were performed for a range of parameter combinations (sensitivity analysis *sensu *[103]) under parsimony. Sensitivity analysis allows discerning between stable relationships (those that are recovered under all or most parameter combinations) and unstable relationships (those that appear only under specific parameter combinations). Twelve parameter combinations (metric transformation step-matrices) were used in the analysis: indel/substitution ratios of 1, 2, and 4; and Tv/Ts ratios of 1, 2, 4, and ∞. When the Tv/Ts cost ratio was other than one, the insertion-deletion cost was set relative to the cost of transversions. Transversions were never given a higher cost than indels. To account for a likely possibility that insertions and deletions spanning multiple contiguous nucleotide positions could have been accommodated by single events, additional sets of parameters taking into account affine indel (gap extension) costs [104] were carried out. These analyses explored the indel-substitution ratios of 1, 2, and 4, and Tv/Ts ratios of 1, 2, 4, where the extension gap cost was set at a constant minimum (half the cost for transitions). This corresponded to five ratios of gap opening/gap extension costs: 2, 4, 8, 16, and 32. In addition, a cost regime that maximizes homology of both sequence fragments and individual nucleotide positions in the analysis of sequences of variable length [105] was also examined. This weighting scheme, based on the view of parsimony as a two-taxon analysis [106], corresponds to the following combination of costs: 2 for substitutions, 3 for gap opening, and 1 for gap extension [105]. In total, 22 parameter combinations were analyzed. The results of the sensitivity analysis are shown as binary plots (Tv/Ts ratio on horizontal and indel/substitution ratio on vertical axes) to designate presence or absence of every clade recovered in the analysis of the complete data set under equal-costs regime. In addition, these results are summarized as a stability tree-a consensus (strict and majority-rule) of clades recovered under the entire parameter space explored. Despite the fact that character and topological congruence, as measured by the ILD [64] and the topological ILD (TILD; [107]) respectively, has been proposed as a criterion for choosing among alternative weighting schemes, these measures are partition-dependent and their values are not comparable across different data matrices [108]. Because of the lack of such an objective criterion to decide among alternative weighting schemes, results from equal costs were preferred. Results under additional parameter value combinations were used to assess clade stability.

## Results

### DNA sequence divergence

A detailed description of DNA sequence divergence patterns is provided in additional file 1: DNA sequence divergence. As expected, the average levels and the range of divergence, based on the estimates of *p *distance (uncorrected for multiple hits), increased with relative taxonomic rank and, when compared across loci, were the smallest for the 18S and largest for the 16S sequences. With regard to the taxonomically problematic *Pinctada imbricata*/*fucata*/*radiata *species complex, even though none of the markers could provide unambiguous cut-off levels for intra- vs. interspecific sequence divergence, in all cases the levels of divergence in pairwise comparisons between *P. imbricata, fucata*, and *radiata *exemplars were invariably lower compared to other interspecific comparisons, and were the same or marginally exceeding the limits of the intraspecific sequence divergence range inferred for other species within *Pinctada*.

### Nucleotide base composition

Overall base composition was homogeneous across all taxa: a χ^2 ^test rejected the null hypothesis of base-composition stationarity neither for any individual locus, nor for the complete data set (Table 3). The 16S sequences had a low (although statistically insignificant) proportion of cytosine and the prevalence of A + T (55.47%), that has been previously reported for other molluscan taxa [45]. Across all loci, the sequences were to some extent GC-rich (A + T = 47.61%, C + G = 52.41%).

**Table 3:** Nucleotide base composition.

Partition	A	C	G	T	**χ**^**2 **^**/*P***
18S	24.96	22.43	27.39	25.22	19.52
	(23.40-25.96)	(21.95-23.47)	(26.65-28.95)	(24.34-25.79)	1.00
28S	21.25	25.83	32.84	20.08	94.78
	(19.58-26.62)	(23.38-27.59)	(31.13-34.04)	(18.24-22.14)	1.00
16S	26.92	17.66	28.55	26.87	196.18
	(23.28-30.46)	(14.81-21.97)	(22.90-32.10)	(22.80-30.86)	0.99
H3	28.76	27.82	24.59	18.83	61.62
	(25.17-30.97)	(25.16-30.98)	(22.58-26.21)	(16.77-21.29)	1.00
All loci	24.29	23.43	28.98	23.32	179.39
	(23.30-26.32)	(22.12-24.68)	(26.44-29.89)	(21.83-25.07)	0.99

The average and range (in parentheses) of base composition (percentage) for each data partition across taxa. The results of the χ^2 ^value (upper) and the *P *value (lower) of the χ^2 ^test (df = 297 for each test) are listed in the rightmost column.

### Patterns of nucleotide substitution

The entropy-based statistical test [52] of the combined molecular data set showed no significant net effect of saturation (Iss < Iss.c; *p *< 0.05). It revealed that the 18S and H3 sequences were unaffected by multiple hits (Iss < Iss.c; *p *< 0.05), but suggested the presence of substantial substitution saturation in the 16S sequence (Iss > Iss.c; *p *< 0.05). Saturation of the 28S sequences can potentially become problematic when extremely asymmetrical topologies are considered (Iss > Iss.cAsym; *p *< 0.05), which are probably unrealistic given the results of the phylogenetic analysis (see below).

The overall value of the shape parameter α of the gamma distribution was 0.51, suggesting extensive heterogeneity in substitution rates across sites. There was a considerable disparity in the levels of α across loci: the highest level of across-site variation was found in 18S (α = 0.37), followed by H3 (α = 0.58), 28S (α = 0.86), and 16S (α = 1.66). It is noteworthy, that all the nuclear loci displayed substantial (L-shaped distributed) variation, whereas the mitochondrial locus showed relatively moderate (bell-shaped distributed) variation in substitution rates among sites. The estimates of α appear to be accurate for such range of α (the standard error is less than 10% of the α value) [109]. In H3 sequences it was likely due to codon bias: virtually no substitutions were inferred for the second position, less than 2% of sites (1.94% Ts and 1.66% Tv) experienced substitutions in the first position, whereas up to roughly 20% (18.33% Ts and 11.95% Tv) of sites in the third position underwent substitution.

### Incongruence-Length Difference Tests

The ILD tests performed simultaneously on all four molecular data partitions showed that the complete data set was homogeneous (*P *= 0.15), suggesting that combining molecular partitions in a phylogenetic analysis was not likely to reduce phylogenetic accuracy. The pairwise ILD tests, revealed, however, that only the rDNA partitions were entirely congruent (18S:28S, *P *= 0.35; 18S:16S, *P *= 0.71; 28S:16S, *P *= 0.99), whereas the H3 data set was congruent with 16S (*P *= 0.15), but not with the nuclear rDNA loci (18S:H3, *P *= 0.01; 28S:H3, *P *= 0.01). Despite the fact that the appropriate threshold of incongruence is not known, it has been argued that a significance threshold of 0.05 might be too conservative for the ILD test [110,111]. Given the overall homogeneity of the combined data and marginal incongruence of H3 data, the data-set incongruence was not likely to render an analysis of the combined data misleading. Consequently, the phylogenetic inference based on the combined data was the preferred description of pterioidean relationships, but one must be cautious when interpreting the results with regard to the contribution of the H3 data. The ILD tests together with phylogenentic analyses of individual data partitions and the assessment of their relative contribution to support on a combined-data cladogram (measured by PBrS values) were used to assess the distribution, nature, and extent of agreement among data sets.

### Combined analyses of molecular data

**Figure 2:**
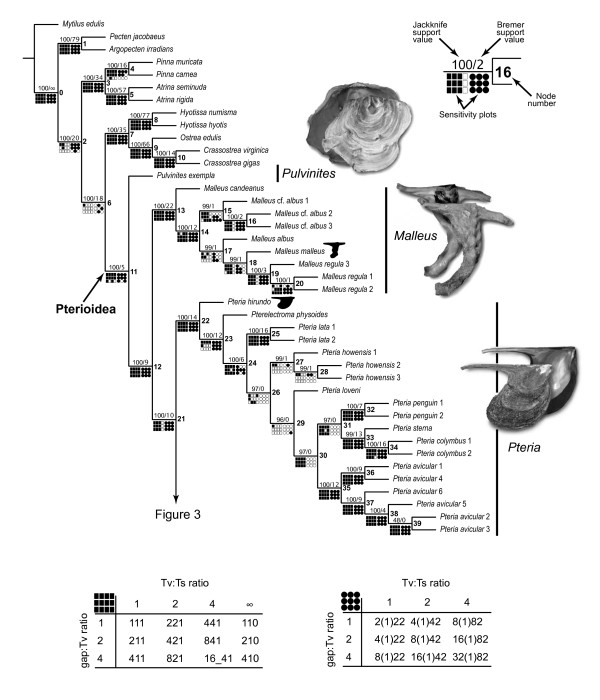
**A partial view of the most-parsimonious tree based on the analysis of the complete data set and the results of the sensitivity analysis showing the relationships of the outgroups, and the genera *Pulvinites*, *Malleus*, and *Pteria***. The single most-parsimonious topology (L = 6027, CI = 0.56, RI = 0.86) of the complete data set resulting from the combined analysis of the 18S, 28S, 16S, and H3 data under uniform weighting. Numbers at the nodes denoted node numbers; numbers above the branches indicate jackknife/Bremer support values. The results of the sensitivity analysis are summarized below the branches as binary plots with filled shapes denoting clade presence and open shapes denoting clade absence. Circles and squares indicate whether the analyses were performed with or without taking the affine gap costs into account respectively. The tables below describe specific parameter combination used for each analysis listing the costs in the following order: "indel/transversion/transition" or "gap opening/(gap extension)/transition/transversion." Shell icons denote type species.

**Figure 3:**
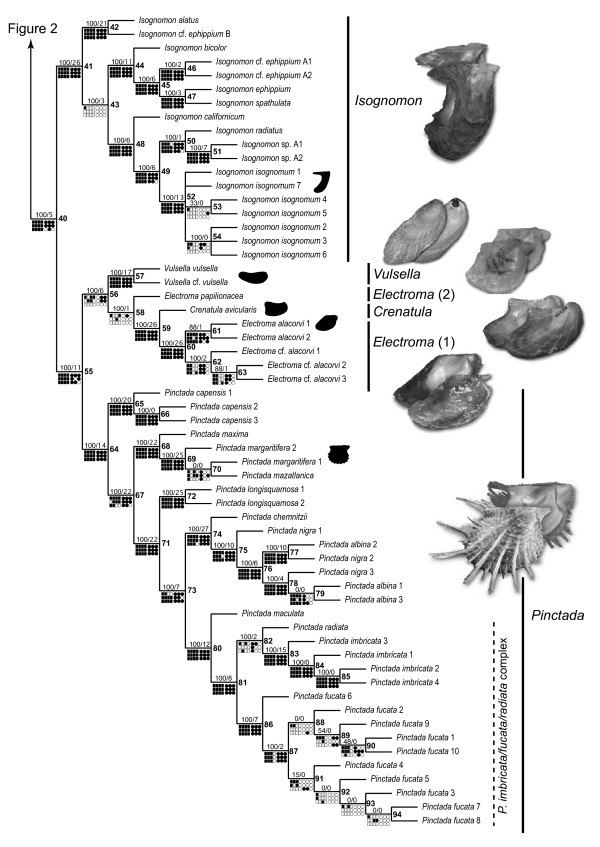
**A partial view of the most-parsimonious tree based on the analysis of the complete data set and the results of the sensitivity analysis showing the relationships of the genera *Isognomon*, *Vulsella*, *Electroma*, *Crenatula*, and *Pinctada***. See Figure 2 for legend.

**Figure 4:**
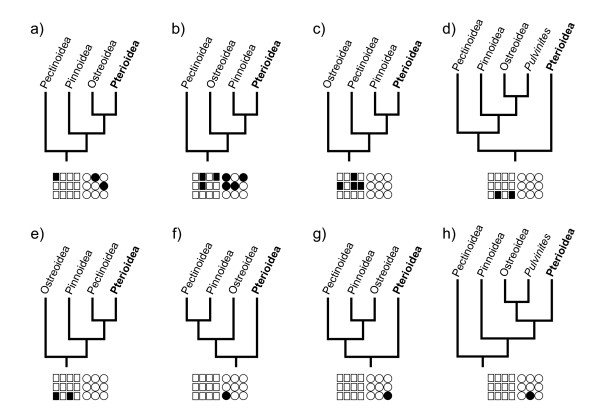
**Alternative hypotheses for the pterioidean sister group**. Each panel represents a summary topology recovered for the combination(s) of alignment parameters indicated by the sensitivity plots below. See Figure 2 for legend.

A parsimony analysis of the complete molecular data set performed under equal-costs regime produced a single, well-resolved optimal cladogram (L = 6027, CI = 0.56, RI = 0.86; Figures 2, 3). The superfamily Pterioidea was recovered as a monophyletic, well-supported clade (JK = 100%; BrS = 5), which was stable under most (92%) combinations of alignment parameters. The few cases of non-monophyly of the superfamily were due entirely to the placement of *Pulvinites exempla *among the outgroup taxa as a sister group to the Ostreoidea (Figures 4d, h). This arrangement was recovered only under parameter combinations characterized by the highest indel/substitution ratio, suggesting that the spurious placement was due to missing data, an expected artifact given the fact that *P. exempla *was the only taxon for which only a small fraction of sequence data was available. Excluding *P. exempla *from the analysis did not affect the relationships of the remaining taxa (1 MPT, L = 5914, CI = 0.55, RI = 0.86), suggesting that the effect of the missing data for this taxon on the relationships among remaining ingroup taxa was negligible.

**Figure 5:**
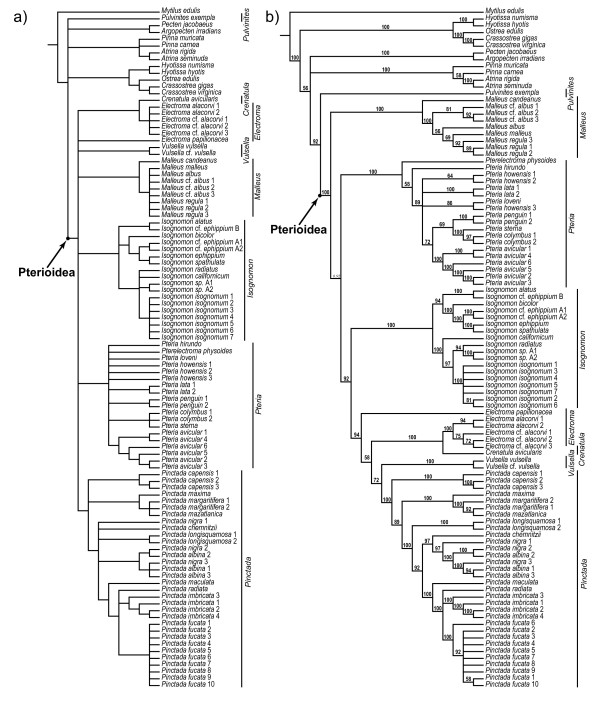
**Stability trees**. Strict (a) and majority-rule (b) consensus trees summarizing cladograms resulting from sensitivity analysis of the complete data set analyzed under 22 alignment parameter combinations (see text for details).

**Figure 6:**
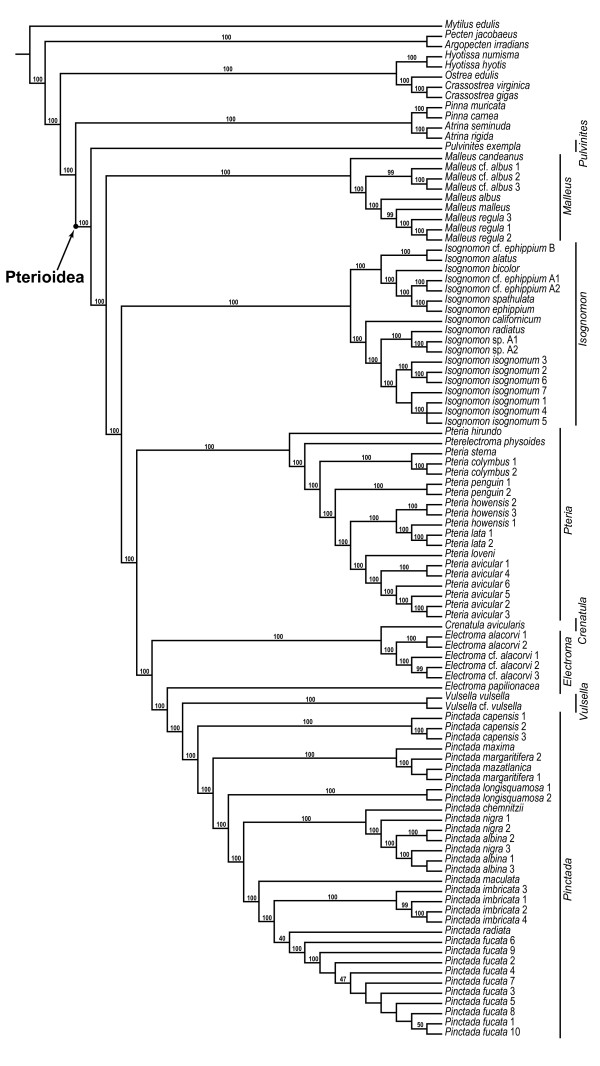
**The most-parsimonious tree based on the analysis of the complete data using non-uniform weighting**. The single most-parsimonious cladogram (L = 12508, CI = 0.24, RI = 0.52) resulting from the combined analysis of the 18S, 28S, 16S, and H3 data under the cost regime that maximizes homology of both sequence fragments and individual nucleotide positions (2 for substitutions, 3 for gap opening, and 1 for gap extension; [105]).

The suprageneric relationships were completely resolved and all polytomies were restricted to a single derived subclade within the *Isognomon *clade. All traditionally recognized families-with the obvious exception of the monotypic Pulvinitidae-were recovered polyphyletic, whereas all genera (except for *Electroma*) were definitively monophyletic. The monophyly of the genera *Pulvinites *and *Crenatula *could not be tested in the present study because these genera were represented by single exemplars. Thus, the polyphyly of the families was a result of unconventional relative placement of traditionally recognized genera. The genus-level clades were distributed in a fully pectinate pattern of relationships with the basal-most *Pulvinites *and the others branching off in the following sequence: *Malleus*, *Pteria*, *Isognomon*, *Pinctada*, *Vulsella*, and *Electroma *(the latter including *Crenatula*, thereby rendering *Electroma *paraphyletic). The nodes along the backbone had relatively high support (JK = 100%; BrS = 5-26) and were stable under the majority of alignment parameters (monophyletic under more than 92% of parameter combinations; Figures 2, 3, 5) with the exception of the *Vulsella*/*Electroma*/*Crenatula *group. Despite the high support values (JK = 100%; BrS = 6) obtained under the equal-costs regime, the monophyly of this clade was recovered in only 41% of alignment parameter combinations. The sister-group relationship of *Crenatula *and *Electroma *(excluding *E. papilionacea*, the placement of which was highly variable) was robust (JK = 100%; BrS = 26) and insensitive to parameter choice. The monophyly of all the genera was very strongly supported (JK = 100%; BrS = 14-26) and entirely stable across alignment parameter space (Figures 2, 3, 5). The monophyly of higher taxa among the outgroups was strongly supported (JK = 100%; BrS = 14-79) in all analyses and was insensitive to the choice of alignment parameters. The Ostreoidea was identified as an immediate outgroup to the Pterioidea with high support (JK = 100%; BrS = 18) under equal-cost regime, but the Pterioidea/Ostreoidea sister-group relationship was highly sensitive to alignment ambiguity and was obtained for only four (18%) combinations of alignment parameters. Effectively, all possible arrangements of the considered superfamilies were recovered (Figure 4). The cladogram generated under the parameter set that maximizes homology of both sequence fragments and base correspondences (1 MPT, L = 12508, CI = 0.24, RI = 0.52; Figure 6), had a similar overall structure to the cladogram generated under uniform weighting with several important differences: (1) the placement of *Isognomon *and *Pteria *clades were exchanged; (2) the *Vulsella*/*Electroma*/*Crenatula *clade was not recovered; and (3) the Pinnoidea was an immediate pterioidean outgroup.

**Figure 7:**
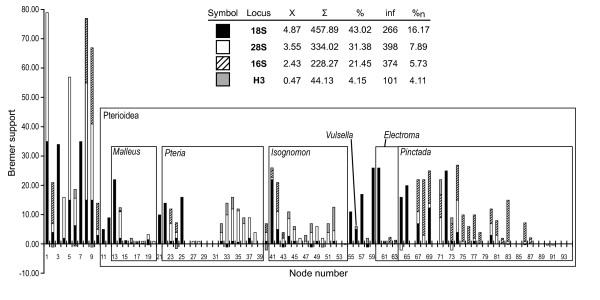
**Partitioned Bremer support (PBrS) analysis**. The histogram shows proportional support of each locus for all the nodes (as labeled in Figure 2). The table above the histogram summarizes the PBrS statistics for each locus. Χ, the average Bremer support; Σ, the total Bremer support; %, the percentage of the cumulative Bremer support; %n, percent normalized by the number of parsimony informative characters.

Relative contributions of different data partitions, calculated as partitioned Bremer support values for each node, are shown in Figure 7. Each locus provides support for some deep and apical nodes, but not all of them. There also appears to be a slight taxonomic bias in the distribution of the PBrS values: for example, relatively high PBrS values for most nodes within *Pteria *were provided by 28S, whereas comparable in magnitude PBrS values for the majority of the nodes within *Pinctada *were provided by 16S. Taken together, 18S and 28S bring close to 75% of the signal and contribute the highest on average support values; H3 provides the least support (4.15%) and the lowest on average support values. When the support of each gene was normalized by the number of informative characters, 18S appeared as most informative, providing more than twice the support as 28S, whereas 16S and H3 were the least informative markers in this data set.

**Figure 8:**
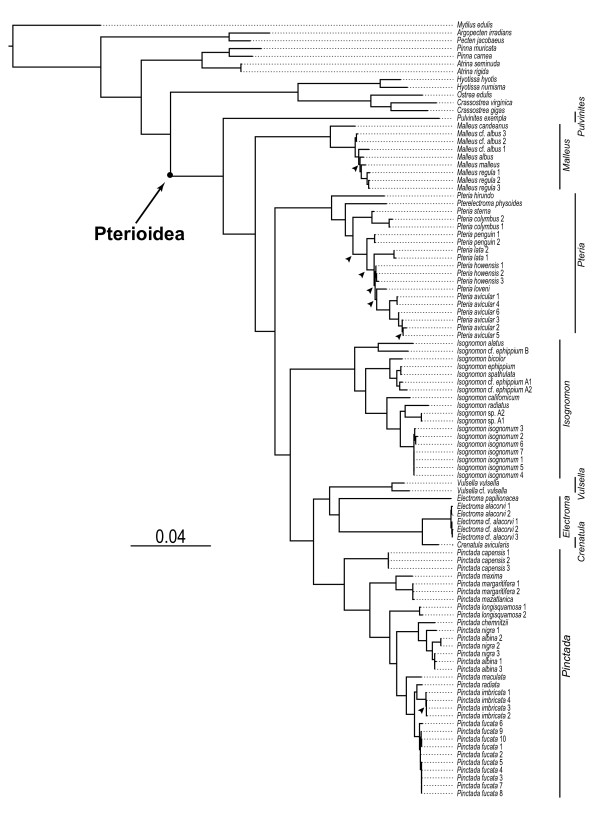
**The maximum likelihood tree resulting from the analysis of the complete data set**. The ML tree (-logLk = 30253.94) resulting from the combined analysis of the 18S, 28S, 16S, and H3 data. The scale bar indicates the number of substitutions per site. The nodes that disagree between the ML and MP topologies are indicated by arrows.

A ML analysis of the complete molecular data set produced in a single optimal tree (-logLk = 30253.94; Figure 8). The values of suboptimal trees from 83.2% of search replicates were within 0.01% of the optimal likelihood value; 11 of these had identical to the optimal likelihood values to the third decimal point and differed only in the placement of several conspecific taxa. The estimated shape parameter α of gamma distribution was 0.22; the substitution relative rate parameters for the model were 0.86 (AC), 2.42 (AG), 1.61 (AT), 0.52 (CG), 4.15 (CT), 1.00 (GT; fixed). Comparison of the optimal parsimony and ML topologies showed non-substantial differences with regard to placement of several species-level taxa within *Pteria *and *Malleus*, and alternate arrangement of conspecific individuals of *Pinctada imbricata.*

**Figure 9:**
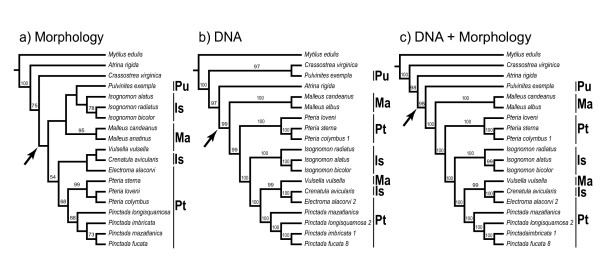
**Parsimony analyses of the reduced data set**. All analyses produced single most-parsimonious trees. For molecular-only and simultaneous analyses the combined data for 18S, 28S, 16S, and H3 data were analyzed under equal-costs alignment regime. (a) Morphological data (length = 317, CI = 0.54, RI = 0.56; data from [15]). (b) The molecular data-only tree (1 MPT, length = 3,094, CI = 70.43, RI = 67,40). (c) Combined molecular and morphological data (1 MPT, length = 3,425, CI = 68.85, RI = 66.14). Numbers above branches are jackknife support values. Arrow designates the Pterioidea. Abbreviations: Is, Isognomonidae; Ma, Malleidae; Pt, Pteriidae; Pu, Pulvinitidae.

A parsimony analysis of the reduced molecular data set performed under equal-costs regime resulted in a single well-supported (JK = 97-100) cladogram (L = 3,094, CI = 70.43, RI = 67.40; Figure 9b). In spite of a great difference in taxon sampling between the complete and reduced data sets, the higher-level relationships were nearly identical with only two notable differences: in the analysis of the reduced data set *Pulvinites exempla *emerged as a sister taxon to the outgroup *Crassostrea virginica *(rendering the Pterioidea polyphyletic) and a pinnid, *Atrina rigida*, was recovered as an immediate outgroup to the Pterioidea. As suggested by the analyses of the complete data set, missing data and sensitivity to alignment ambiguity coupled with the limited taxon sampling of the reduced data set probably account respectively for the instability in the position of *P. exempla *and the placement of the immediate outgroup.

The phylogenetic analyses of individual molecular data partitions resulted in cladograms that differ in topology, degree of resolution, and relative support measures. However, all the analyses invariably recovered the Pterioidea and major genus-level clades monophyletic. (For detailed discussion of partitioned analyses of molecular data see additional file 2: Partitioned analyses.)

### Combined analyses of morphological and molecular data

A parsimony analysis of the combined morphological and the reduced molecular data sets (hereafter referred to as the "combined reduced data set") performed under equal-costs regime produced a single, highly supported tree (L = 3425, CI = 68.85, RI = 66.14; Figure 9c). When compared to the tree generated for the reduced (molecular-only) data set analyzed under the same conditions, the addition of morphological data reconstituted monophyletic the Pterioidea by placing *Pulvinites exempla *at the base of the pterioidean subtree; the placement of the remaining taxa was identical. Similarly, the arrangements of pterioidean genera in the analyses of the combined reduced and complete molecular data sets were identical with the only significant topological difference being the identity of the immediate pterioidean sister-group: the Pinnoidea in the former and the Ostreoidea in the latter.

The paired-sites tests, applied to three topologies constrained to the identical arrangement of pterioidean taxa (corresponding to that obtained in the analysis of the combined reduced data set under uniform weighting), but differing solely in the placement of the outgroup taxa, showed that the compared trees were not significantly different (*P *> 0.05; Table 4). Only the monophyly of the Ostreoidea-Pinnoidea clade could be rejected in one set of tests under parsimony (KH test, *P *= 0.0243; Templeton test, *P *= 0.0243).

**Table 4:** Paired-sites tests of alternative hypotheses of pterioidean sister group.

Alternative topologies		Paired-sites tests P values		
**H_1_**	**H_2_**	**KH (ML)**	**Templeton**	**KH (MP)**

Pin(Pte/Ost)	Ost(Pte/Pin)	0.526	0.3248	0.3248
Pin(Pte/Ost)	Pte(Pin/Ost)	0.081	0.0243*	0.0243*
Ost(Pte/Pin)	Pte(Pin/Ost)	0.200	0.1985	0.1986

The significance of the length difference between alternative hypotheses of relationships (H_1 _and H_2_) were evaluated using the Kishino-Hasegawa (KH) test under parsimony (MP) and maximum likelihood (ML), and the Wilcoxon signed-ranks test (Templeton). * = significant difference, P < 0.05. Other abbreviations: Ost, Ostreoidea; Pin, Pinnoidea; Pte, Pterioidea.

**Figure 10:**
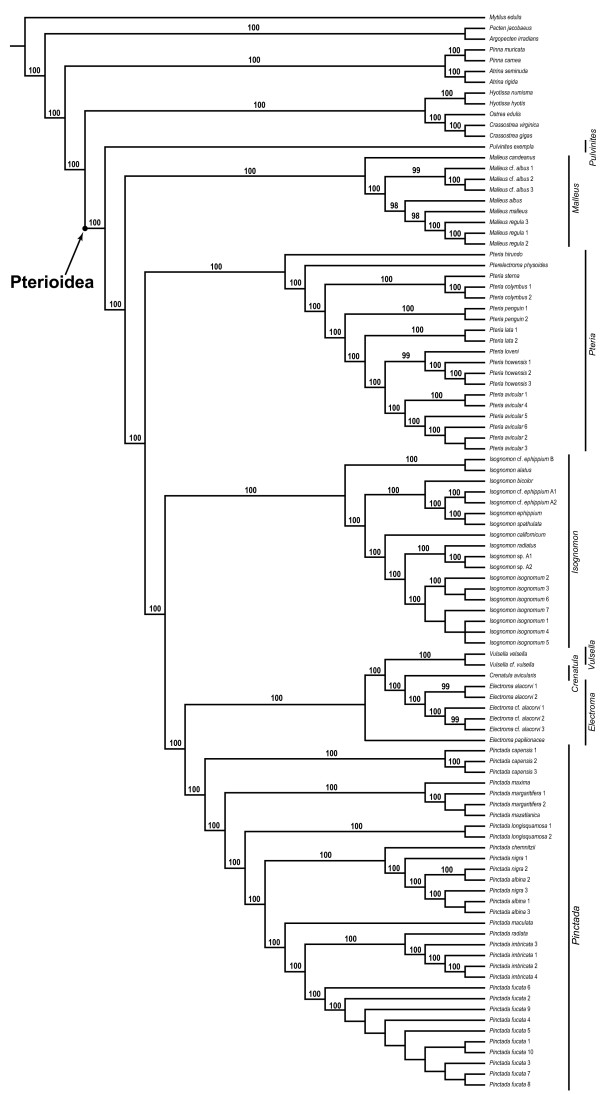
**Parsimony analysis of the complete combined data set**. The single most-parsimonious tree (L = 6358, CI = 56, RI = 86) resulting from the parsimony analysis of combined molecular (18S, 28S, 16S, and H3) and morphological data under uniform weighting. Numbers above branches are jackknife support values.

A parsimony analysis of the combined morphological and the complete molecular data sets (hereafter referred to as the "combined complete data set") produced a well-resolved and strongly supported single optimal cladogram (L = 6358, CI = 56, RI = 86; Figure 10). The topology was essentially the same as of the tree obtained under the same conditions for the complete molecular-only data set: the higher-level relationships along the backbone were identical. The few differences included an alternative placement of several clades within *Pteria *(that had extremely low Bremer support values and were unstable in the sensitivity analysis) and the rearrangement of several, probably conspecific, representatives of *Isognomon*. The most significant discrepancy between the analyses was the placement of *Electroma papilionacea*. In the combined analysis it was recovered at the base of the (*Vulsella*(*Electroma*/*Crenatula*)) clade, whereas in the molecular-only study it was nested within it: (*Vulsella*(*E. papilionacea*(*Electroma*/*Crenatula*))).

## Discussion

### Reliability of phylogenetic inference

The present study provides the first explicit hypothesis for higher-level phylogeny and species-level relationships for the bivalve superfamily Pterioidea based on DNA sequence data.

Nearly complete species-level taxon and character coverage suggest that the random error associated with sampling was not likely to compromise the fidelity of the results inasmuch as supraspecific relationships are concerned. Uncertainty in species-level relationships does, however, remain, due to incomplete taxon sampling, differences in character sampling among congeneric individuals, and potentially insufficient phylogenetic signal of chosen markers for resolving recent divergences. Sequence alignment was straightforward and unambiguous for the protein-coding gene. For the ribosomal sequences, the effect of the uncertainty in nucleotide positional homology on stability of inferred relationships was evaluated using the sensitivity analysis, which allowed for recognizing specific parts of the phylogeny affected by the uncertainty in nucleotide homology and evaluating the extent of its effect.

Nucleotide base composition was nearly perfectly stationary across taxa overall as well as for individual loci, without evidence of taxon attraction due to similar nucleotide-composition bias (e.g., [112,113]).

Substitution saturation in DNA sequences can potentially underestimate the actual amount of evolutionary change and reduce the phylogenetic signal due to excessive multiple hits in the same nucleotide positions resulting in extensive homoplasy (e.g., [52]). Despite the fact that extensive saturation was detected for transitions in the 16S sequence, its effect on the phylogenetic inference was negligible, as shown by (1) comparison of the results from individual data partitions, (2) incorporating the Ts/Tv into phylogenetic analysis, (3) character congruence among data partitions, and (4) consistency in the distribution of BrS values. Furthermore, excluding 16S data from the analysis resulted in a decreased resolution and support throughout the tree. These results are consistent with theoretical and empirical findings suggesting that homoplasy does not necessarily confound phylogenetic inference [114-117] and that a given site, which is homoplastic in a sub-part of the most-parsimonious tree, can bring phylogenetic information for another sub-part of the same tree [118].

Heterogeneity in substitution rates among sites within a nucleotide sequence can have considerable impact on sequence divergence [56,119-121]. Substantial heterogeneity in substitution rates among lineages resulting in long-branch attraction is another source of misleading inference in molecular data [122,123]. The agreement between the results of the maximum likelihood analysis under the model allowing for variable base frequencies and parsimony suggested that the predominance of transitions and among-site heterogeneity for most loci, as well as among-lineage heterogeneity in nucleotide substitution rates (implied by the differences in branch lengths) were not likely to be a source of misleading inference. The absence of long-branch attraction despite considerable differences in branch lengths was consistent with prior observation that extensively sampled phylogenies are not sensitive to this artifact [124,125].

The doubly uniparental inheritance (DUI) [126], which has been reported for a number of bivalve species [127], can potentially affect the accuracy of phylogenetic reconstruction due to varying degrees of divergence and homogenization between conspecific genomes. Within the subclass Autolamellibranchiata, the instances of DUI were reported only from the most basal extant group, Mytiloida. Even though the lack of reported instances of DUI in a particular group does not necessarily imply that it is not present, the absence of strict dioeciousness in the Pterioidea suggests that DUI was not a likely to be a problem. All pterioidean species for which reproductive strategies have been elucidated (mostly members of the genera *Pinctada *and *Pteria*), are rhythmical protandrous hermaphrodites, with occasional instances of protogyny and simultaneous hermaphroditism, and mtDNA appears to be maternally inherited [128].

Simultaneous analysis of a single data matrix combining independent character sets potentially increases the descriptive efficiency and explanatory power [129], provides the basis for evaluating the relative support and taking into account the hidden support provided by individual partitions [130], and is less sensitive to sampling bias [131]. However, the incongruence among data partitions may indicate that they have evolved under different models of evolution and render a combined-data analysis misleading [132]. Despite topological differences obtained in the analyses of individual data partitions, the results of ILD tests showed that the data sets were largely congruent, implying that a single phylogeny underlies the relationships inferred from individual data partitions. Differences in substitution rates across sites, the average pairwise sequence divergence values, the distribution and levels of support (expressed in PBrS values), and the extent of resolution obtained in the analyses of individual partitions indicated that the marker loci collectively encompassed sufficiently broad spectrum of DNA sequence divergence to produce a nearly completely resolved and well-supported tree. These results also suggested that topological differences among data partitions were probably due to characters that evolved rapidly to provide a reliable signal for sub-terminal (recent) nodes of a tree, but produced noise for the resolution of deeper (ancient) nodes. The overall increased resolution and support of the combined-data trees when compared to the results of partitioned analyses strongly speak for the simultaneous analysis approach.

In summary, the present molecular sequence data and the choice of methodology adequately account for sampling biases and potentially confounding (for phylogenetic inference) effects of various molecular evolutionary processes, providing a strong foundation for accurate, reliable, and robust reconstruction of phylogenetic relationships of the Recent Pterioidea.

### Phylogeny of the Pterioidea

The present study corroborates the monophyly of the superfamily Pterioidea and definitively rejects that of all nominal pterioidean families (with the exception of the monotypic Pulvinitidae). Whereas the non-monophyly of some traditional pterioidean groups was anticipated by prior studies [15,17-19], the arrangement of the genera was novel and unexpected, as well as resolved and strongly supported.

In terms of taxonomic congruence with morphological studies, the analysis of molecular data offered a considerably different phylogenetic hypothesis. Generally assumed close relatedness of commercially important genera *Pteria *and *Pinctada *was rejected by the molecular evidence, which was not entirely surprising given the absence of unambiguously optimized non-homoplastic characters supporting the sister-group relationship of these taxa [15]. The presumed close relatedness of the genera *Malleus *and *Isognomon*, implied by superficial resemblance in shell morphology and similar life habits [133-135], was also contradicted by the molecular data. The only morphological character supporting a close relatedness of these two genera-the absence of food grooves on inner demibranchs of the gills-is symplesiomorphic in the context of the DNA based topology, as this condition has been also reported from other autolamellibranchiate taxa (e.g., Pectinidae and Limidae; [136]). Contrary to previously proposed hypothesis [15,137], this study rejected a close affinity of *Isognomon *and *Pulvinites*, placing the latter at the base of the pterioidean tree. This finding was corroborated by the fact that in the morphology-based analysis (Figure 9a) the sister-group relationship of the two genera was weakly supported and relied on a unique combination of shared homoplastic characters (presence of the multivincular ligament, absence of the pallial fold, and serial tissue fusion of the distal edges of the demibranchs).

The present analysis provided the first molecular evidence for a peculiar clade, recently recognised on morphological grounds (Figure 9a) [15], that consists of relatively understudied, low-diversity genera *Electroma*, *Vulsella*, and *Crenatula*, that were traditionally placed in Pteriidae, Malleidae, and Isognomonidae respectively. The arrangement among the genera, however, varied between the two studies. In the morphology-only analysis, where each genus was represented by a single exemplar, *Electroma *was basal to *Vulsella *and *Crenatula*, the sister-group relationship between which was implied by the reduction of the pedo-byssal apparatus. Instead, in the molecular analysis, *Vulsella *appeared basal to the *Electroma *clade that nested the sole representative of *Crenatula*, rendering *Electroma *paraphyletic. An unanticipated discovery of polyphyletic *Electroma *requires detailed morphological and taxonomic reappraisal of this poorly known genus. Another species of *Electroma*, typically placed in its own subgenus *Pterelectroma*, *E*. (*P.*) *physoides*, invariably grouped with the *Pteria *clade, putting into question the taxonomic status of this subgenus.

Due to high sensitivity to alignment parameters and taxon sampling, uncertainty remains regarding the identity of the sister group of the Pterioidea. The paired-sites tests, applied to the alternative topologies differing only in the placement of the outgroup taxa, showed that the trees were not significantly different. Consequently, the sister-group of the Pterioidea could not be unequivocally inferred in light of the present results. Based on the results of the combined-data analyses under uniform weighting and predominant support (in terms of the number of non-homoplastic morphological apomorphies), the Ostreoidea is tentatively proposed here as the most likely pterioidean sister group.

### Implications for homology of morphological characters

It has long been acknowledged that extensive homoplasy is a recurrent theme in bivalve phenotypic evolution [138-142] that can potentially mislead phylogenetic inference based on morphological data alone. Optimizing morphological character state transformations onto topology derived from the analysis of molecular data provides means to discern putative homologies and instances of homoplasy. The molecular analyses presented here have confirmed a number of suspected convergences identified on the basis of morphology, but also uncovered a number of hitherto unanticipated ones. The hypotheses of homology of several key characters (that have been considered phylogenetically significant in the Pterioidea as well as in other bivalves) were evaluated in light of a robust phylogenetic hypothesis based on molecular evidence.

A duplicated outer fold of the mantle edge (a "four-fold" condition) has been considered a derived trait that evolved twice independently in the Pterioidea based on the analysis of morphological data [15] and in agreement with presumably "primitive" three-fold arrangement [143-146]. In contrast, molecular data suggested that the four-fold condition was primitive in the Pterioidea, whereas the three-fold condition was a result of a single reversal to the three-fold condition exhibited by the outgroup taxa. This finding was not entirely unexpected, given the fact that analogous transitions in mantle evolution had occurred in other bivalve lineages [15].

In the analysis of morphological data, homorhabdic ctenidia (simple gills characterized by a single type of filaments) evolved once from heterorhabdic ctenidia (that contain ordinary and apical filaments). Molecular results, on the other hand, implied that the gill grades had evolved iteratively and the ancestral condition could not be unequivocally resolved. The presence of food grooves in the outer demibranchs showed the same pattern.

The loss of postlarval byssus and posterior pedo-byssal retractor muscles has been previously considered as evidence for sister-group relationship of *Vulsella *and *Crenatula *[15]. Both features are likely to be adaptations for living inside sponges, the habit shared by members of the two genera, where physical stabilization is achieved by the attachment to the host sponge. The relationships inferred from the molecular data invariably grouped *Crenatula *with *Electroma*, a relationship supported by a single unambiguously optimized nonhomoplastic apomorphy: a T-shaped (in cross-section) typhlosole, a longitudinal fold of the intestine. Molecular results were ambiguous with regard to the immediate relatedness of the *Vulsella *and the *Crenatula/Electroma *clades due to alignment uncertainty, and suggested a possibility that the evolution of adaptation for living inside sponges could have been two separate events. This supposition is corroborated by prior ecological observations that the host specificity, and the nature of mutualistic association of *Vulsella *and *Crenatula *species with their demosponge hosts are different: the former is a facultative mutualist with keratosid sponges, whereas the latter is an obligatory mutualist with monaxonid sponges [147,148]. Species of *Vulsella *possess a relatively thick and elongated shell with an extremely abbreviated hinge line and a narrow prismatic margin that provide a rigid "endo-skeleton" for the sponge, hence facilitating the host's growth. Species of *Crenatula*, on the other hand, have a very thin shell with a very wide prismatic margin, which appears to be influenced by the sponge growth, and produce highly irregular shell shapes without consistent orientation. The particular form of the multivincular ligament of *Crenatula *allows for curvature of the extensive hinge line, which facilitates attachment along the hinge line to irregular surfaces. The loss of postlarval byssus in distantly related *Malleus albus *(not included in the analysis of morphological data) also supports the likelihood of convergence.

The symmetry and relative placement of abdominal sense organs (ASOs), paired mechanoreceptors typically found on the surface of the adductor muscle, have been previously shown to be phylogenetically informative within Autolamellibranchiata [149,150] and, specifically, within the Pterioidea [15]. In the morphology-based analysis, the clustering of the ASOs around the anus was inferred as a primitive condition in the Pterioidea (found in both ostreid and pinnid outgroup taxa), whereas the dorsal displacement of the right ASO had occurred twice. According to the results of the molecular character analysis, both aspects of the ASO position had evolved twice.

A set of characters that has historically been used for higher-level systematics of pterioidean and other autolamellibranchiate taxa is associated with the types of ligament and their support structures. According to the molecular-based topology, the multivincular ligament characterizes three distantly related genera: *Pulvinites*, *Isognomon*, and *Crenatula*. Morphological data alone also identified an independent origin of multivincular ligament in *Crenatula*, but suggested a sister-group relationship of *Pulvinites *and *Isognomon *on the basis of shared ligament morphology and two other homoplastic characters. It is noteworthy that despite apparent similarity between multivincular systems in *Pulvinites *and *Isognomon*, the pattern of ligostracum deposition and the ontogenetic sequence of origination of ligamental layers along the hinge line differ between the genera. Even though not entirely congruent, the results of the molecular and morphological studies reject the long-standing assumption of close relatedness based on a single origin of multivincular ligament in the Pterioidea.

The present molecular evidence confirmed the taxonomic significance of the shape of the anal funnel, an ear-shaped membranous structure situated at the posterior extremity of the rectum that presumably facilitates expulsion of fecal pellets. The absence of the anal funnel in *Pulvinites *is consistent with its basal placement in the Pterioidea as the same condition is displayed by the outgroup taxa. In contrast, in the analysis based on morphological data, the lack of the funnel was inferred as a loss. The molecular and morphological analyses agree that the anal funnel is subtriangular in outline in the more basal pterioidean clades, whereas it is characteristically lanceolate and rounded in two most derived clades, *Pinctada *and *Vulsella*/*Crenatula/Electroma*, respectively.

Given the uncertainty in the identity of pterioidean sister group, it is instructive to explore the effect of alternative combinations of outgroup taxa on the distribution of diagnostic morphological characters using the best available hypothesis of the ingroup relationships. To this end, morphological characters were optimized on three trees constrained to the identical arrangement of the ingroup taxa (corresponding to that obtained in the analysis of the combined reduced data set under uniform weighting) but differing in the placement of the outgroups.

The Pinnoidea(Ostreoidea/Pterioidea) relationship (L = 330, CI = 0.52, RI = 0.53) was defined by five unambiguously optimized, non-homoplastic apomorphic characters that remained unchanged above this node: (1) the descending intestine produced towards the posteroventral side of the posterior adductor muscle; (2) the loss of the anterior adductor muscle (monomyary); (3) the absence of the pseudonymphae; (4) the presence of a resilifer throughout ontogeny; and (5) the placement of the posterior adductor muscle entirely within the boundary of the inner shell layer. Previously, Steiner and Hammer [18] had mapped a set of morphological characters on to their maximum likelihood tree substantiating their finding of ostreoidean/pterioidean sister-group relationship by two characters: the monomyary and the presence of the anal funnel. The present study confirmed the monomyaryan condition as a synapomorphy of the clade, but not the occurrence of the anal funnel in different superfamilies, interpreted here as a result of independent origins, as was previously suggested on the basis of morphological data [15,22]. The Ostreoidea(Pinnoidea/Pterioidea) relationship (L = 334, CI = 0.51, RI = 0.52) was defined by three apomorphic characters: (1) the absence of the marginal mantle lobe fusion; (2) the absence of the eulaterofrontal cirri of gill filaments; and (3) the ascending intestine not produced anteriorly passed the stomach. The Pterioidea(Ostreoidea/Pinnoidea) relationship (L = 333, CI = 0.52, RI = 0.52) was defined by two apomorphic characters: (1) the absence of the ciliated discs on gill filaments and (2) the clustering of ducts of the digestive diverticula. Given comparable levels of homoplasy and the lack of *a priori *criteria to differentially weigh the characters supporting each of the possibilities, the arrangement supported by the largest number of apomorphies and yielding the shortest tree length, that is (Pinnoidea(Ostreoidea/Pterioidea)), is the preferred hypothesis of relationship.

### Species-level taxonomy of *Pinctada*

Despite the fact that the focus of the present study was to establish higher-level relationships within the Pterioidea, it provided significant insights into species-level taxonomy of the genus *Pinctada*, a commercially important and the most exhaustively sampled group.

The present study suggests that *Pinctada imbricata*, *P*. *fucata*, and *P. radiata *are distinct genealogical units. The taxonomic status of the *P. imbricata*/*fucata*/*radiata *species complex previously remained unsettled due to extensive variation in shell characters within and among populations, extremely wide geographical distribution, and transport and hybridization by humans [32]. Traditionally, three distinct species were recognized corresponding to three biogeographic realms: *P. imbricata *in the western Atlantic region, *P. radiata *in the eastern Indian Ocean and the Red Sea regions, and *P. fucata *in the Indo-Pacific region. The Japanese populations have frequently been regarded as a distinct species, *P. martensii*, or a subspecies, *P. fucata martensii*. Because of its major role in perliculture, the species complex had become a major research focus in recent years. A large number of comparative genetic analyses using allozyme and DNA sequence data indicated significant levels of gene flow over large geographic distances among populations of Indian Ocean and Indo-Pacific regions, resulting in low levels of genetic differentiation [8,32]. The pattern of genetic differentiation among the Chinese, Japanese, and Australian populations using amplified fragment length polymorphism (AFLP) markers was consistent with isolation by distance [151,152]. Shell morphometric studies [153,154], mating experiments [155], and comparative karyotype analyses [156-158] failed to establish unequivocal species delineations. To date, no diagnostic discrete morphological characters have been identified for populations from the three geographical regions.

The present results showed shallow levels of divergence among the members of the *P. imbricata*/*fucata*/*radiata *species complex, comparable to the levels of divergence of conspecific exemplars of other *Pinctada *species. However, the three groups were reciprocally monophyletic showing an apparent clinal geographical structure. Therefore, it might be meaningful to treat these individual populations as evolutionary significant units (ESUs). Taking into account the comparatively low divergence values and the lack of diagnostic morphological characters distinguishing these groups, from the taxonomic standpoint it is advocated here to provisionally recognize them at a subspecies level under the senior synonym, *P. imbricata *(as *Pinctada imbricata imbricata*, *P*. *imbricata fucata*, and *P*. *imbricata radiata*). The final word on the taxonomic status of these taxa will depend on the future population-level analysis with dense sampling throughout the entire distribution range and using genetic markers more sensitive for resolving recent divergencies.

This study also suggested that *Pinctada albina *and *P. nigra*, generally distinguished by shell color (as their names imply), represent two colormorphs of the same species. This possibility has been previously suggested based on extremely low levels of genetic divergence between two exemplars of *P. albina *and one of *P. nigra *[151]. This finding was consistent with other molecular studies that invariably resolved these species (represented by single exemplars) as sister taxa [159-161]. The present analysis contained three exemplars of each colormorph that were always recovered as a monophyletic group and never formed separate clades diagnosed by color.

In addition, based on the fact that *P. mazatlanica *invariably nested among representatives of *P. margaritifera *or members of both species formed an unresolved clade, the present study provides preliminary support for conspecificity of these two species. These findings need to be investigated further and cannot be definitively tested given the present sample size.

## Conclusions

The most significant conclusion from the phylogenetic analyses presented here is that the state of pterioidean systematics is no longer in chaos. The present results establish a solid-however unconventional- framework for the higher-level taxonomy of the group, focusing future revisionary systematic effort and providing a better grasp of standing alpha-diversity. The phylogeny is crucial for understanding processes and patterns of diversification and morphological evolution of the Pterioidea through time. To this end, the major future challenge lies in integrating the knowledge on the Recent pterioideans with information on the extensive, well-documented fossil record of the group. As throughout their history, pterioideans remain a globally distributed, relatively common group of bivalves found in most warm, shallow marine environments around the globe, and are likely to display patterns of diversity typical of other marine benthic macrofauna. Therefore, the insights gained from the phylogenetically-informed studies of pterioidean bivalves might be broadly applicable across marine invertebrates.

## Supplementary Material

Additional file 1 **DNA sequence divergence**. Word DOC file containing DNA sequence divergence. Click here for file

Additional file 2 **Partitioned analyses**. Word DOC file displaying Partitioned analyses. Click here for file
