# Supplementary material for: Molecular phylogeny of pearl oysters and their relatives (Mollusca, Bivalvia, Pterioidea)
Source: BMC Evol Biol. 2010 Nov 8;10:342. doi: 10.1186/1471-2148-10-342 (PMC3271234; doi:10.1186/1471-2148-10-342)
Supplement: Additional file 1 — DNA sequence divergence. Word DOC file containing DNA sequence divergence. [file 1471-2148-10-342-S1.DOC]

**DNA Sequence Divergence**

Average DNA sequence divergence values (*p* distance uncorrected for multiple hits) for intra- and intergeneric comparisons between the ingroup taxa per locus are shown in Tables 1 and 2 respectively. The average levels of divergence averaged across taxa for within- and among-genus comparisons as well as for the ingroup-outgroup comparisons greatly varied across the loci and increased in the following order: 18S, 28S, H3, and 16S (Table 3). The range of sequence divergence values also increased in the same progression for inter- and intra-generic comparisons, but in the ingroup-outgroup comparisons the 28S sequence had most disparity. Despite differences in their values, the range and the magnitude of sequence divergence among loci were constantly larger for intergeneric than for intrageneric comparisons. Also, the relative divergence levels within and among genera (measured as the ratio of the average intergeneric to intrageneric divergence values) were similar across loci and differed by approximately a factor of 3.7. The disparity of intergeneric and intrageneric divergence was most pronounced in the 18S (5.97) and the least—in the H3 (2.72) sequences, whereas the LSU sequences had intermediary values (28S: 3.40; 16S: 2.86). For all loci, the average sequence divergence values for ingroup—outgroup pairs of taxa were higher than the divergences among the ingroup genera, which, in turn, were higher than the divergences within the ingroup genera.

The levels of average pairwise sequence divergence among congeneric exemplars differed among pterioidean genera (Table 1). The divergence values for the nuclear rDNA were the highest within *Electroma* (18S: average *p* = 1.38; 28S: average *p* = 4.87), whereas the values for the other loci were the highest within *Isognomon* (16S: average *p* = 19.34; H3: average *p* = 6.37). The genus *Malleus* had the least amount of divergence for all nuclear markers [average *p* range = 0.12% (18S)—1.69% (H3)], whereas the genus *Electroma* showed least amount of divergence for the mitochondrial ribosomal sequence (average *p* = 0.34). The levels of average interspecific divergence values were roughly comparable among genera for any given locus, with a notable exception of 16S, which showed exceptionally low divergence for *Electroma* (<1%), whereas the divergence among species in other genera were in the range of 8-13% for this gene fragment.

Additional comparisons based on minimum-evolution distances were carried out among representatives of *Pinctada* for evaluating the extant of sequence divergence for the taxonomically problematic *Pinctada imbricata*/*fucata*/*radiata* species complex (Table 4). In *Pinctada*, the average within-species divergence in 18S sequences was extremely low, 0.06% (range 0-0.17), whereas the average among-species divergence was an order of magnitude higher, 0.53% (range 0-1.82). The levels of variation within *P. imbricata* (0%) and *P. fucata* (0.03%) (variation within *P. radiata* could not be obtained because it was represented by a single exemplar) were essentially the same as when compared among them (*fucata/radiata*: 0%; *imbricata/radiata*: 0.01%; *fucata/imbricata*: 0.01%), well within the relative limit of the range of intraspecific variation of other species of *Pinctada*. However, the same low levels of divergence were also identified for all comparisons between representatives of *P. imbricata*/*fucata*/*radiata* and a closely related *P. maculata*, ranging from 0 to 0.01%. In the analysis of 28S sequences, the total average of interspecific pairwise divergence values was 2.35% (range 0.33-4.90%), whereas the intraspecific divergence values were below 0.5%, 0.20% (total range 0-0.32%). The average divergence values for inter- and intraspecific comparisons of *P. imbricata*/*fucata*/*radiata* were 0.21% (range 0.1-0.31%) and 0.08% (range 0-0.16%) respectively, both at or below the average for intraspecific divergence values within the genus. In contrast to the 18S data, the levels of divergence in all comparisons of *P. imbricata*/*fucata*/*radiatai* with *P. maculata* were substantially higher, ranging from 1.00% to 1.21%. It is noteworthy, that, in similar fashion, the average divergence values between *P. margaritifera* and *P. maxima* were approximating the intraspecific level of divergence for the 28S locus (0.33%) but clearly exceeded it for 18S (0.16%). As expected, the largest disparity between inter- and intraspecific divergence values was shown by the mitochondrial 16S sequences: the total average of interspecific pairwise divergence values was 17.50% (range 7.77-25.11%), whereas the intraspecific divergence was 1.17% (range 0-3.50%). The average divergence values for inter- and intraspecific comparisons of *P. imbricata*/*fucata*/*radiata* were 5.73% (range 5.03-6.70%) and 0.18% (range 0.10-0.25%) respectively, the latter somewhat exceeding the average intraspecific divergence level inferred for other congeners.

**Table 1**. Average pairwise DNA sequence divergence values (percent uncorrected *p* distance) and transition-transversion ratios in pterioidean genera for four loci. The numbers in parentheses refer to the number of pairwise comparisons.

|  | *Locus* | *Malleus* | *Pteria* | *Isognomon* | *Pinctada* | *Vulsella* | *Electroma* |
| --- | --- | --- | --- | --- | --- | --- | --- |
| *P* | 18S  28S  16S  H3 | 0.12 (36)  1.08 (28)  7.96 (10)  1.69 (36) | 0.35 (171)  3.53 (171)  12.57 (15)  3.19 (171) | 0.40 (153)  2.31 (153)  19.34 (10)  6.37 (153) | 0.43 (676)  2.47 (496)  13.61 (465)  5.10 (120) | 0.79 (1)  1.47 (1)  –  – | 1.38 (10)  4.87 (10)  0.34 (10)  3.74 (15) |
| Ts/Tv | 18S  28S  16S  H3 | 1.08 (36)  3.72 (28)  2.41 (10)  2.31 (36) | 1.36 (171)  2.97 (171)  1.37 (15)  3.20 (171) | 1.08 (153)  1.81 (153)  1.48 (10)  2.18 (153) | 3.25 (676)  2.37 (496)  2.25 (465)  1.69 (120) | 0.75 (1)  1.29 (1)  –  – | 1.23 (10)  1.72 (10)  1.25 (10)  0.68 (15) |

**Table 2**. Average pairwise DNA sequence divergence values (percent uncorrected *p* distance above the diagonal) and transition-transversion ratios (below the diagonal) among pterioidean genera. Each cell contains values for 18S, 28S, 16S, and H3 loci in that order. The numbers in parentheses refer to the number of pairwise comparisons.

| Genus | *Electroma* | *Crenatula* | *Isognomon* | *Malleus* | *Pinctada* | *Pteria* | *Pulvinites* | *Vulsella* |
| --- | --- | --- | --- | --- | --- | --- | --- | --- |
| *Electroma* | –  –  –  – | 0.78 (5)  3.55 (5)  13.67 (5)  3.82 (6) | 3.56 (90)  8.83 (90)  33.49 (25)  12.70 (108) | 3.72 (45)  10.35 (40)  34.98 (25)  10.74 (54) | 3.44 (160)  8.31 (160)  32.29 (155)  11.19 (96) | 3.39 (95)  11.60 (95)  30.93 (30)  9.20 (114) | 5.23 (5)  –  –  – | 2.94 (10)  8.93 (10)  –  11.11 (6) |
| *Crenatula* | 0.34 (5)  1.34 (5)  2.07 (5)  1.51 (6) | –  –  –  – | 3.65 (18)  7.96 (18)  33.54 (5)  11.68 (18) | 3.76 (9)  9.56 (8)  33.09 (5)  10.24 (9) | 3.51 (32)  7.35 (32)  31.92 (31)  11.05 (16) | 3.47 (19)  10.63 (19)  31.75 (5)  8.96 (19) | 5.11 (1)  –  –  – | 3.00 (2)  8.04 (2)  –  11.65 (1) |
| *Isognomon* | 0.93 (90)  1.47 (90)  1.25 (25)  1.78 (108) | 0.92 (18)  1.41 (18)  1.26 (5)  1.55 (18) | –  –  –  – | 2.47 (162)  8.86 (144)  32.56 (25)  13.79 (162) | 2.83 (576)  7.65 (576)  30.51 (155)  12.41 (288) | 1.84 (342)  9.95 (342)  28.21 (30)  12.30 (342) | 5.14 (18)  –  –  – | 2.66 (36)  8.10 (36)  –  14.18 (18) |
| *Malleus* | 1.15 (45)  1.21 (40)  1.13 (25)  1.10 (54) | 1.08 (9)  1.23 (8)  1.19 (5)  0.94 (9) | 1.70 (162)  1.46 (144)  1.04 (25)  1.42 (162) | –  –  –  – | 3.18 (288)  9.30 (256)  33.17 (155)  10.76 (144) | 1.84 (171)  10.85 (152)  31.38 (30)  10.05 (171) | 5.03 (9)  –  –  – | 3.29 (18)  9.50 (16)  –  11.04 (9) |
| *Pinctada* | 1.01 (160)  1.61 (160)  0.99 (155)  1.66 (95) | 0.94 (32)  1.78 (32)  1.04 (31)  1.24 (16) | 1.18 (576)  1.91 (576)  1.15 (155)  1.51 (288) | 1.51 (288)  1.65 (256)  1.00 (155)  1.28 (144) | –  –  –  – | 2.68 (608)  10.20 (608)  30.45 (186)  11.05 (304) | 5.19 (32)  –  –  – | 2.77 (64)  6.81 (64)  –  10.36 (16) |
| *Pteria* | 1.04 (95)  1.50 (95)  1.08 (30)  1.89 (114) | 1.05 (19)  1.44 (19)  1.17 (5)  1.94 (19) | 1.00 (342)  1.54 (342)  0.90 (30)  1.85 (342) | 1.57 (171)  1.44 (152)  1.08 (30)  1.80 (171) | 1.26 (608)  1.54 (608)  0.88 (186)  1.78 (304) | –  –  –  – | 4.60 (19)  –  –  – | 2.89 (38)  11.01 (38)  –  12.08 (19) |
| *Pulvinites* | 0.99 (5)  –  –  – | 0.95 (1)  –  –  – | 1.08 (18)  –  –  – | 1.13 (9)  –  –  – | 1.00 (32)  –  –  – | 0.98 (19)  –  –  – | –  –  –  – | 4.82 (2)  –  –  – |
| *Vulsella* | 1.05 (10)  1.36 (10)  –  1.77 (6) | 1.04 (2)  1.56 (2)  –  1.25 (1) | 0.82 (36)  1.36 (36)  –  2.02 (18) | 1.24 (18)  1.28 (16)  –  1.44 (9) | 0.74 (64)  1.41 (64)  –  2.54 (16) | 0.77 (38) 1.38 (38)  –  2.06 (19) | 0.80 (2)  –  –  – | –  –  –  – |

**Table 3**. The average levels of DNA divergence (percent uncorrected *p* distance) for within- and among-genus, and ingroup-outgroup comparisons.

|  | 18S | 28S | 16S | H3 |
| --- | --- | --- | --- | --- |
| Among genera | 3.46 (0.78-5.23) [28] | 8.92 (3.54-11.60) [21] | 30.80 (13.67-34.98) [15] | 10.97 (3.82-14.18) [21] |
| Within genera | 0.58 (0.12-1.38) [6] | 2.62 (1.08-4.87) [6] | 10.76 (0.34-19.34) [5] | 4.02 (1.69-6.37) [5] |
| Ingroup-outgroup | 6.18 (3.87-8.46) [1045] | 14.09 (3.92-23.24) [1020] | 38.80 (32.29-46.82) [684] | 16.04 (11.11-20.65) [630] |

**Table 4**. Average pairwise DNA sequence divergence values (calculated using the GTR model) within and among the species of *Pinctada*.

| Species | *capensis* | *margaritifera* | *maxima* | *longisquamosa* | *chemnitzi* | *albina* | *maculata* | *imbricata* | *radiata* | *fucata* |
| --- | --- | --- | --- | --- | --- | --- | --- | --- | --- | --- |
| *capensis* | 0 (3)  0 (3)  0 (3) | 0.45 (9)  3.84 (9)  25.11 (9) | 0.53 (3)  3.49 (3)  23.85 (3) | 0.65 (6)  4.83 (6)  22.51 (3) | 0.88 (3)  4.79 (3)  24.19 (3) | 1.82 (18)  4.84 (18)  25.06 (18) | 0.82 (3)  4.89 (3)  20.82 (3) | 0.84 (12)  4.80 (12)  23.16 (12) | 0.82 (3)  4.69 (3)  23.59 (3) | 0.82 (30)  4.90 (30)  23.01 (30) |
| *margaritifera* |  | 0.08 (3)  0.27 (3)  0 (3) | 0.16 (3)  0.33 (3)  11.26 (3) | 0.28 (6)  2.51 (6)  21.65 (3) | 0.57 (3)  1.74 (3)  22.08 (3) | 0.62 (18)  1.94 (18)  22.98 (18) | 0.45 (3)  2.04 (3)  18.70 (3) | 0.47 (12)  1.74 (12)  20.01 (12) | 0.45 (3)  1.84 (3)  18.66 (3) | 0.45 (30)  1.77 (30)  18.05 (30) |
| *maxima* |  |  | --  --  -- | 0.35 (2)  2.41 (2)  21.91 (1) | 0.65 (1)  1.80 (1)  22.34 (1) | 0.71 (6)  2.02 (6)  23.87 (6) | 0.54 (1)  2.10 (1)  20.04 (1) | 0.54 (4)  1.80 (4)  20.71 (4) | 0.53 (1)  1.70 (1)  20.00 (1) | 0.53 (10)  1.81 (10)  18.74 (10) |
| *longisquamosa* |  |  |  | 0 (1)  0.20 (1)  -- | 0.41 (2)  2.61 (2)  13.71 (1) | 0.56 (12)  2.49 (12)  14.68 (6) | 0.41 (2)  2.41 (2)  12.71 (1) | 0.43 (8)  1.81 (8)  15.06 (4) | 0.41 (2)  1.91 (2)  13.74 (1) | 0.41 (20)  2.03 (20)  13.57 (10) |
| *chemnitzi* |  |  |  |  | --  --  -- | 0.27 (6)  0.77 (6)  10.04 (6) | 0.59 (1)  1.90 (1)  12.42 (1) | 0.60 (4)  1.50 (4)  13.08 (4) | 0.59 (1)  1.60 (1)  13.24 (1) | 0.59 (10)  1.63 (10)  12.39 (10) |
| *albina* |  |  |  |  |  | 0.17 (15)  0.32 (15)  3.50 (15) | 0.48 (6)  1.81 (6)  13.26 (6) | 0.49 (24)  1.37 (24)  13.15 (24) | 0.48 (6)  1.47 (6)  13.24 (6) | 0.48 (60)  1.50 (60)  12.97 (60) |
| *maculata* |  |  |  |  |  |  | --  --  -- | 0.01 (4)  1.00 (4)  9.51 (4) | 0 (1)  1.10 (1)  7.77 (1) | 0 (10)  1.21 (10)  8.03 (10) |
| *imbricata* |  |  |  |  |  |  |  | 0.03 (6)  0 (6)  0.10 (6) | 0.01 (4)  0.10 (4)  5.46 (4) | 0.01 (40)  0.23 (40)  6.70 (40) |
| *radiata* |  |  |  |  |  |  |  |  | --  --  -- | 0 (10)  0.31 (10)  5.03 (10) |
| *fucata* |  |  |  |  |  |  |  |  |  | 0 (45)  0.16 (45)  0.25 (45) |
