# Supplementary material for: Molecular phylogeny of pearl oysters and their relatives (Mollusca, Bivalvia, Pterioidea)
Source: BMC Evol Biol. 2010 Nov 8;10:342. doi: 10.1186/1471-2148-10-342 (PMC3271234; doi:10.1186/1471-2148-10-342)
Supplement: Additional file 2 — Partitioned analyses. Word DOC file displaying Partitioned analyses. [file 1471-2148-10-342-S2.DOC]

# Partitioned analyses

A comparison among cladograms obtained for different partitions is complicated by the fact that they do not share exactly the same complement of taxa but some general observations can nevertheless be drawn. In all analyses of rDNA sequences the Pterioidea was recovered monophyletic with high support (JK > 99; Fig. 1). In the H3 cladogram, on the other hand, *Electroma papilionacea* and the entire *Malleus* clade nested among the outgroup taxa, however with weak support (JK < 33). Consistent with the results of the combined analysis, all partitioned analyses confirmed non-monophyly of pterioid families and supported the monophyly of all the genera except for *Electroma* due to an ambiguous position of *E. papilionacea.* The latter was found either basal to the (*Vulsella*(*Crenatula*(*Electroma*)) clade (18S), basal to the rest of the Pterioidea (28S), or nested among the outgroup taxa (H3). The alternate placements of *E. papilionacea* among the ingroup taxa were well supported (JK > 99), whereas its exclusion from the Pterioidea was not (JK < 33). The relative arrangement of pterioidean genera varied greatly across the analyses with the 16S tree being entirely consistent with the topology obtained in the analysis of the combined data. (It is noteworthy, that the 16S locus was neither the most informative in terms of the number of parsimony-informative characters, nor contributed the highest levels of support.) The alternative relationships along the backbone were well supported (JK = 98-100) in the analyses of the rDNA data, but extremely weakly supported (JK = 15-45) in the analysis of H3 data. The most unconventional relationships were recovered in the analysis of the H3 data (non-monophyly of the Pterioidea and the placement of a *Vulsella* representative among exemplars of *Pinctada*), but they had exceptionally low support values (JK = 15-33). Moreover, the H3 data had the highest extent of homoplasy as expressed by the lowest CI and RI values relative to the values obtained for the ribosomal sequence data sets. The level of resolution, expressed by the number of polytomies, also varied across partitions increasing in the following order: 18S, 28S, H3, and 16S. In all partitioned analyses, the unresolved relationships were found exclusively among congeneric exemplars and primarily involved the apical nodes.

Given the potentially confounding transition saturation in the 16S data, the contribution of the mitochondrial partition and its effect on the topology of the combined analysis was evaluated by comparing the results from parsimony analysis of (1) the complete data set, (2) the nuclear DNA data partition (18S, 28S, and H3), (3) the 16S data partition under uniform weighting, and (4) the 16S data partition with upweighted transitions. The analysis of the combined nuclear sequence data produced two optimal trees (L = 3387, CI = 57.56, RI = 88.18). The consensus topology (Fig. 2a) was similar to the tree generated under the same conditions with the mitochondrial data included, had comparable levels of support, but showed somewhat diminished resolution at subterminal nodes. The principal topological difference resulting from the exclusion of the 16S partition was the sister-group relationship between the *Malleus* and *Pteria* clades (also recovered in the analysis of the 28S partition). The topology obtained for the 16S partition under equal weighting was identical to that of the combined data set with regard to the arrangement of pterioidean genera along the backbone but differed from it by favoring Pinnoidea as an immediate outgroup to the Pterioidea (Fig. 1c).

To take into account the actual Ts/Tv ratio, the absolute number of substitutions for the 16S data set was inferred by optimizing both classes of character changes on the optimal topology. The numbers of unambiguously optimized transitions and transversions for 16S were 570 and 464 respectively, and the Ts/Tv ratio = 1.23. With ambiguously optimized substitutions included, the average numbers of transitions and transversions were 1065.02 and 916.99 respectively, and the Ts/Tv ratio = 1.16. Considering the empirically obtained Ts/Tv ratio (which agrees with a general observation that transitions occur twice as frequently as transversions in most eukaryote nuclear genomes; [1]), a parsimony analysis of the mitochondrial partition, was performed with transversions weighted twice transitions. Such differential weighting of character-state changes to reflect the observed frequency of the different types of transformations were shown to substantially improve the performance of phylogenetic methods, especially at high rates of change [2]. The tree recovered under weighted parsimony had essentially the same and somewhat better resolved topology (1 MPT, length = 4000, CI = 40.76, RI = 77.29) than the tree obtained under equal weighting but with overall weaker support (Fig. 2b). It also placed *Pinctada capensis* as basal species of *Pinctada*, which agreed with the results obtained for individual partitions and the combined data set. The analysis of the partitioned Bremer support results indicated that the support provided by the 16S sequence data was consistent with the support provided by nuclear markers.

# References

1. Graur D, Li W-H: **Fundamentals of molecular evolution**, 2 edn. Sunderland, Mass: Sinauer; 2000.

2. Hillis DM, Huelsenbeck JP, Cunningham CW: **Application and accuracy of molecular phylogenies**. *Science* 1994, **264**(5159):671-677.

| 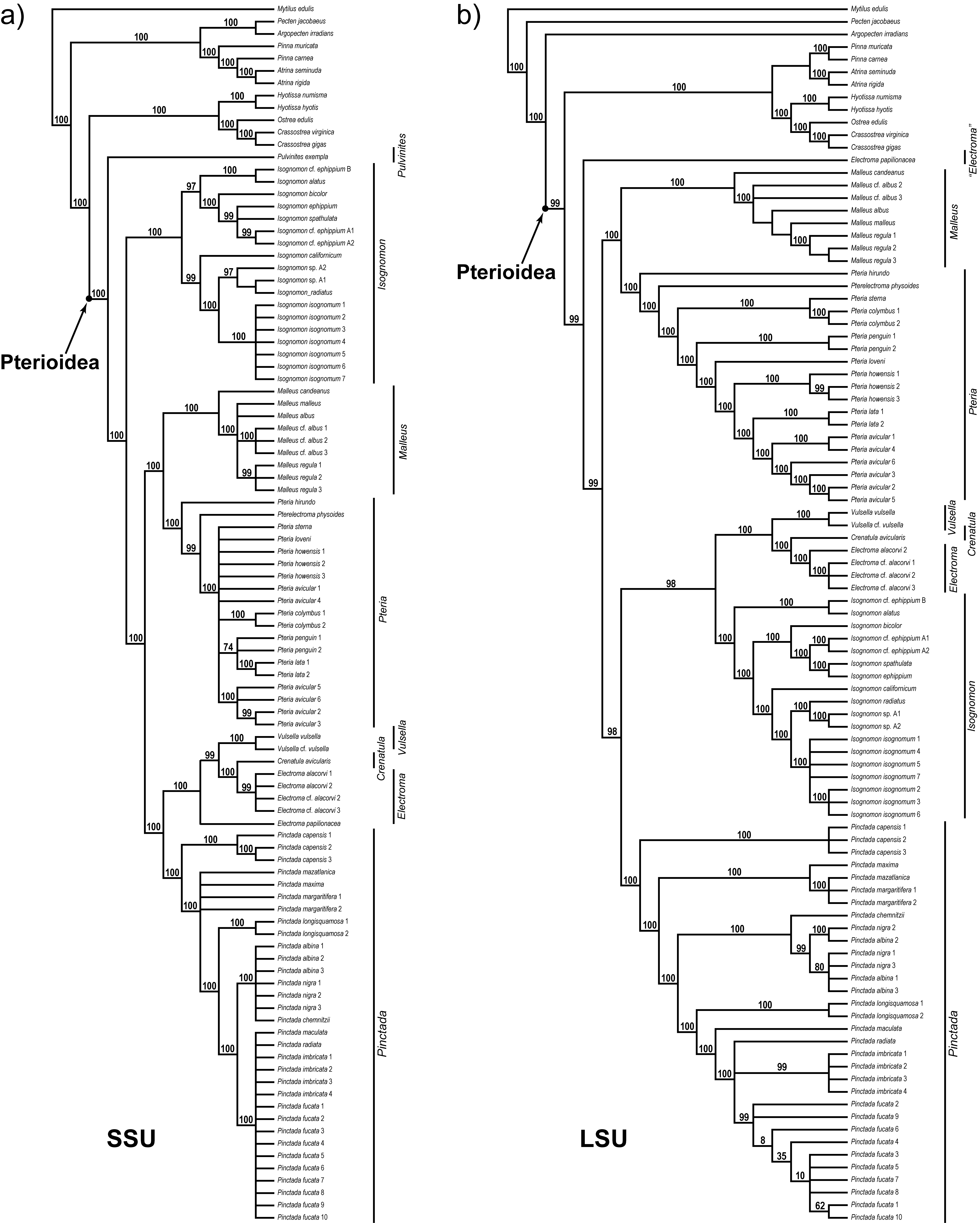 |
| --- |
| **Figure 1 - Parsimony analyses of partitioned molecular data under equal costs regime**  (a) 18S rDNA (strict consensus of 12 MPTs, L = 944, CI = 68.33, RI = 91.92); (b)  28S rDNA (1 MPT; L = 1807, CI = 61.21, RI = 89.44); (c) 16S rDNA (1 MPT, L = 2599, CI =  54.23, RI = 82.81); (d) H3 (strict consensus of three MPTs, L = 555, CI = 29.91, RI = 79.01).  Numbers above branches denote jackknife support values. |
| 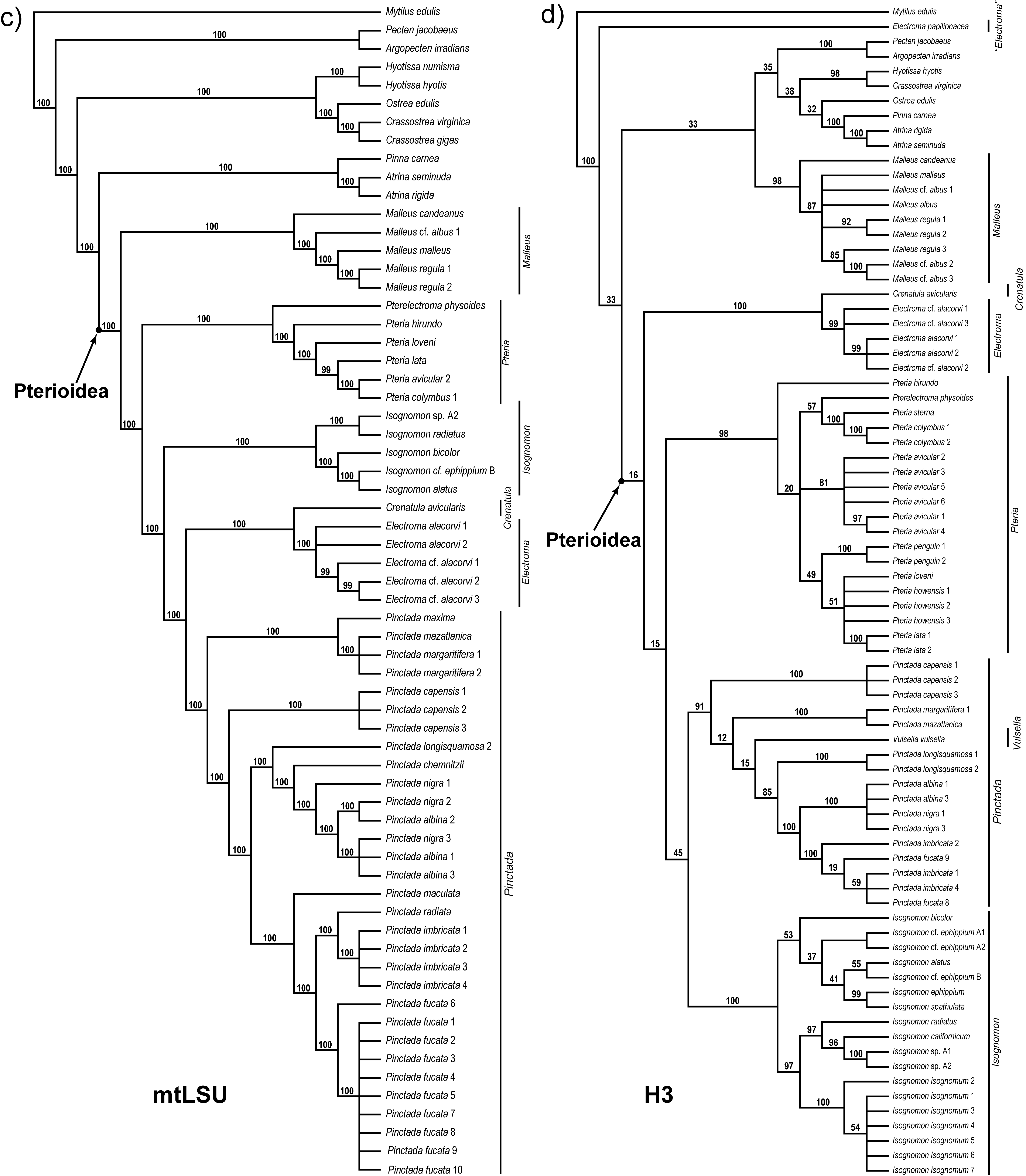 |
| **Figure 1 -** *Continued.* |
| 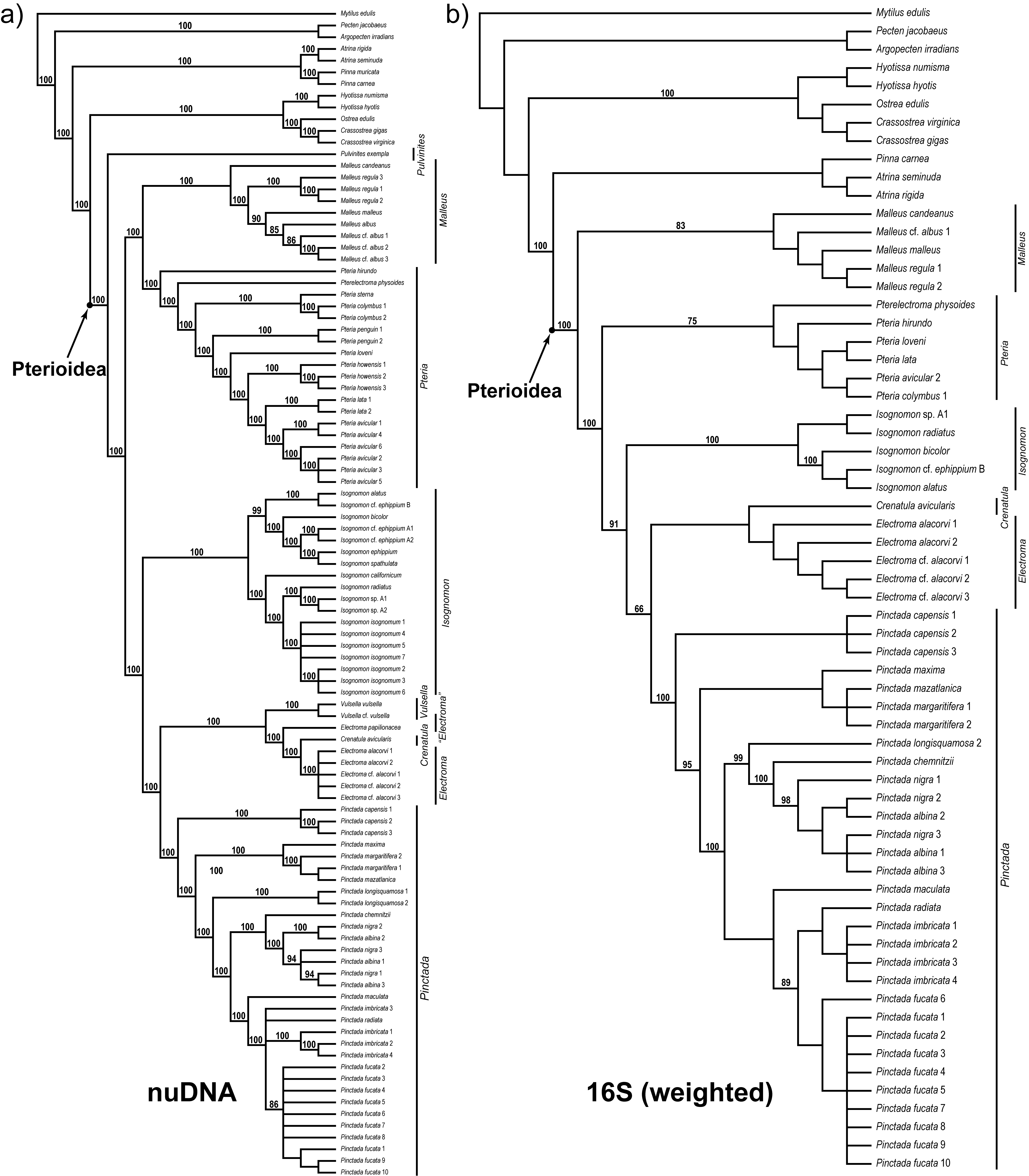 |
| **Figure 2 - Evaluating the effect of the mitochondrial partition**  (a) Parsimony analysis of the nuclear DNA partition (18S, 28S, and H3) data under equal-costs regime. A strict consensus of two optimal trees (L = 3387; CI = 57.56, RI = 88.18). (b) Cladogram obtained for mitochondrial partition (16S) under weighted parsimony with transversions weighted twice transitions (1 MPT, length = 4000). Numbers above branches denote jackknife support values. |
